# Lipophilic statins inhibit YAP nuclear localization, co-activator activity and colony formation in pancreatic cancer cells and prevent the initial stages of pancreatic ductal adenocarcinoma in *Kras^G12D^* mice

**DOI:** 10.1371/journal.pone.0216603

**Published:** 2019-05-17

**Authors:** Fang Hao, Qinhong Xu, Jing Wang, Shuo Yu, Hui-Hua Chang, James Sinnett-Smith, Guido Eibl, Enrique Rozengurt

**Affiliations:** 1 Department of Medicine, David Geffen School of Medicine at UCLA, Los Angeles, California, United States of America; 2 Tianjin Medical University, Tianjin, China; 3 Xi’an Jiaotong University, Xi'an, China; 4 Department of Surgery, David Geffen School of Medicine at UCLA, Los Angeles, California, United States of America; 5 CURE: Digestive Diseases Research Center, Los Angeles, California, United States of America; 6 VA Greater Los Angeles Health Care System, Los Angeles, California, United States of America; University of Texas Health Science Center at San Antonio, UNITED STATES

## Abstract

We examined the impact of statins on Yes-associated Protein (YAP) localization, phosphorylation and transcriptional activity in human and mouse pancreatic ductal adenocarcinoma (PDAC) cells. Exposure of sparse cultures of PANC-1 and MiaPaCa-2 cells to cerivastatin or simvastatin induced a striking re-localization of YAP from the nucleus to the cytoplasm and inhibited the expression of the YAP/TEAD-regulated genes *C*onnective *T*issue *G*rowth *F*actor (*CTGF)* and Cysteine-rich angiogenic inducer 61 (*CYR61*). Statins also prevented YAP nuclear import and expression of *CTGF* and *CYR61* stimulated by the mitogenic combination of insulin and neurotensin in dense culture of these PDAC cells. Cerivastatin, simvastatin, atorvastatin and fluvastatin also inhibited colony formation by PANC-1 and MiaPaCa-2 cells in a dose-dependent manner. In contrast, the hydrophilic statin pravastatin did not exert any inhibitory effect even at a high concentration (10 μM). Mechanistically, cerivastatin did not alter the phosphorylation of YAP at Ser^127^ in either PANC-1 or MiaPaCa-2 cells incubated without or with neurotensin and insulin but blunted the assembly of actin stress fiber in these cells. We extended these findings with human PDAC cells using primary KC and KPC cells, (expressing KrasG12D or both KrasG12D and mutant p53, respectively) isolated from KC or KPC mice. Using cultures of these murine cells, we show that lipophilic statins induced striking YAP translocation from the nucleus to the cytoplasm, inhibited the expression of *Ctgf*, *Cyr61* and *Birc5* and profoundly inhibited colony formation of these cells. Administration of simvastatin to KC mice subjected to diet-induced obesity prevented early pancreatic acini depletion and PanIN formation. Collectively, our results show that lipophilic statins restrain YAP activity and proliferation in pancreatic cancer cell models *in vitro* and attenuates early lesions leading to PDAC *in vivo*.

## Introduction

Pancreatic ductal adenocarcinoma (PDAC), the most common form of pancreatic cancer, is one of the most fatal human diseases, with an overall 5-year survival rate of only 7%. It has been anticipated that PDAC will be the 3^rd^ leading cause of cancer-related mortalities in the USA by the end of 2018 [1] and projected to become the 2^nd^ leading cause of cancer-related mortality in the USA before 2030 [2]. Consequently, new approaches for the treatment and prevention of PDAC are urgently needed.

PDAC arises from precursor lesions, the most common of which are pancreatic intraepithelial neoplasia (PanIN) [3, 4]. Virtually all PanIN lesions and invasive PDAC entail activating mutations in the *KRAS* oncogene, which represent an initiating event in the development of the disease [5, 6]. In line with this concept, the model that best recapitulates the progression of human PDAC in mice involves expression of a mutant *Kras* (Kras^G12D^) from the endogenous *Kras* locus [7]. Administration of an obesogenic diet markedly accelerates PanIN formation and PDAC development in this model [8, 9]. The identification of novel targets and agents for prevention and interception [10] requires a detailed understanding of the signaling mechanisms and gene regulatory programs that stimulate the proliferation of PDAC cells [7].

Recent evidence indicates that the transcriptional co-activators Yes-Associated Protein (YAP) and WW-domain-containing Transcriptional co-Activator with PDZ-binding motif (TAZ), two central effectors of the highly conserved Hippo pathway [11–13], act as potent oncogenes in PDAC [14–17] and in the control of differentiation of pancreatic cells to different lineages [18]. The Hippo pathway consists of a serine/threonine kinase cascade in which Mst1/2 kinases phosphorylate and activate Lats1/2, which phosphorylate YAP and TAZ at specific residues that regulate their localization and protein stability [11, 12]. In its unphosphorylated state, YAP localizes to the nucleus where it binds and activates predominantly the TEA-domain DNA-binding transcription factors (TEAD 1–4) thereby stimulating the expression of multiple genes, including *C*onnective *T*issue *G*rowth *F*actor (*CTGF)* and Cysteine-rich angiogenic inducer 61 (*Cyr61*). Independently of the Hippo pathway, YAP/TAZ localization and activity is highly responsive to the organization of the actin filament [13]. Consequently, YAP/TAZ activity represents a key point of convergence in the signaling by Ras, Rho, tyrosine kinase receptors, G protein-coupled receptors (GPCR), integrins, mechanical cues and cell density, all of which regulate the organization of the actin cytoskeleton and play a critical role in PDAC development [13, 19–23]. Several studies indicate that YAP/TAZ is up-regulated in PDAC tumor samples [24–26] and higher expression of YAP is correlated with poorer survival in PDAC patients [17, 27]. Furthermore, amplification and overexpression of Yap can substitute for mutant Kras expression in preclinical models of PDAC [24]. In view of all these findings, there is intense interest in targeting YAP/TAZ to prevent or intercept PDAC development.

Although inhibition of the activity of transcription factors or their co-activators has proven a difficult strategy, recent evidence suggests a new avenue to target YAP/TAZ activity via the use of statins in PDAC and other malignancies. Many studies showed that the mevalonate pathway is markedly upregulated in several epithelial cancers, including PDAC [28–31]. Statins are specific inhibitors of the 3-hydroxy-methylglutaryl (HMG) CoA reductase [32, 33], the rate-limiting enzyme in the generation of mevalonate, the first step in the biosynthesis of isoprenoids, leading to farnesyl pyrophosphate (FPP), geranylgeranyl pyrophosphate (GG-PP) and cholesterol [34]. The transfer of the geranylgeranyl moiety of GG-PP to a COOH-terminal cysteine of Rho GTPases is critical for their membrane localization and function in signal transduction through actin remodeling [12]. Accordingly, YAP and TAZ act as novel sensors of mevalonate metabolism and statins as inhibitors of their nuclear localization and transcriptional activity, at least in some cell types [35–37]. Statins, which are usually well tolerated and generally safe, have long been used to treat hypercholesterolemia and prevent cardiovascular diseases. Recent epidemiological studies imply that use of statins is associated with beneficial effects in PDAC [38–47], and other malignancies [48]. In a previous preclinical study, statin administration delayed PDAC in mice harboring Kras^G12D^ but the incidence of PDAC in this report was unusually high and the connection between statin and YAP activity was not examined [49]. Furthermore, the impact of statins on the initial stages of PDAC, namely depletion of pancreatic acini (acinar-ductal metaplasia) and formation of PanIN lesions induced by an obesogenic diet in conditional Kras^G12D^ (KC) mice remains unknown.

In the present study, we determined the impact of different statins on YAP localization, and transcriptional activity in human and murine PDAC cells. In order to determine whether statins act via stimulation of the Hippo pathway, we also examined the influence of statins on YAP phosphorylation at Ser^127^, a residue targeted by LATS1/2. Subsequently, we assessed the impact of simvastatin administration on pancreatic acini depletion and PanIN formation using KC mice subjected to diet-induced obesity (DIO), which accelerates PanIN formation and PDAC development [8, 9, 50]. Our results show that exposure of PDAC cells to lipophilic statins, including cerivastatin and simvastatin, inhibits YAP nuclear localization, transcriptional activity and colony formation via a phosphorylation-independent mechanism. Administration of simvastatin to KC mice subjected to DIO attenuated early pancreatic acini depletion and PanIN formation. Collectively, our results provide preclinical evidence that statins restrain YAP activity and proliferation in pancreatic cancer cell models *in vitro* and attenuates early lesions leading to PDAC *in vivo*

## Material and methods

### Cell culture

The human pancreatic cancer cell lines PANC-1 and MiaPaCa-2 were obtained from the American Type Culture Collection (ATCC, Manassas, VA). These cell lines were chosen because they harbor mutations typical of human pancreatic cancer, including mutations in *KRAS* and *TP53* (encoding the p53 protein) and deletion of *CDKN2A* (also known as p16 or p16^INK4a^). These cell lines, authenticated by ATCC by short-tandem repeat analysis, were used within 15 passages and cultured for less than 6 months after recovery from frozen stocks (no authentication was done by the authors). PANC-1 and Mia PaCa-2 cells were grown in Dulbecco's modified Eagle Medium (DMEM) with 2 mM glutamine, 1 mM Na-pyruvate, 100 units/mL penicillin, and 100 μg/mL streptomycin and 10% fetal bovine serum (FBS) at 37°C in a humidified atmosphere containing 10% CO_2_.

Primary KC and KPC cells, (expressing KrasG12D or both KrasG12D and mutant p53, respectively) isolated from KC or KPC mice, were a kind gift from Dr. Ashok Saluja, (Professor and Vice-Chair, Department of Surgery, Director, Sylvester Pancreatic Cancer Research Institute, University of Miami). KC and KPC cells were validated by PCR analysis of genomic DNA. These cells were cultured in RPMI 1640 medium with 2 mM glutamine, 100 units/mL penicillin, and 100 μg/mL streptomycin and 5% fetal bovine serum (FBS) at 37°C in a humidified atmosphere containing 5% CO_2_.

### Experimental animals

After weaning, offspring of *LSL-KrasG12D/+* and *p48-Cre+/−* mice were randomly allocated to a control diet (CD), a high fat, high calorie diet (HFCD), or a HFCD plus simvastatin (40mg/kg). All mice had free access to the diet, body weights were measured weekly and the general health and behavior of animals were assessed daily. After 14 weeks, mice were euthanized and the entire pancreas was harvested for histological analysis. All studies involving animals were reviewed and approved by the Chancellor’s Animal Research Committee of the University of California, Los Angeles in accordance with the NIH Guide for the Care and Use of Laboratory Animals (protocol number: 2011–118)

### Genotyping analysis

*LSL-KRASG12D* and *Cre* alleles were detected prior to randomizing to the experimental diets by PCR analysis of genomic DNA, as described [51]. Mutant (KC) mice expressed both *LSL-KRASG12D* and *Cre* alleles, and animals carrying neither allele were labeled wild type (WT).

### Experimental diets

Diets were prepared by Dyets, Inc. (Bethlehem, PA). At one month of age, mice were randomized to receive either the CD, HFCD, or HFCD with simvastatin (40mg/kg) from Sigma-Aldrich, (St. Louis, MO) in the food. A detailed composition of the diets was described previously [9]. Briefly, while the CD contained 12% calories from fat, 40% of calories in the HFCD stem from fat (corn oil-based). All diets were stored at −20°C (long-term) or 4°C (short-term) in sealed containers and prepared under low light conditions. The diets were replaced on a weekly basis.

### Pancreas and liver histology

Hematoxylin and eosin (H&E) stained tissue sections of the pancreas, fixed in formalin and embedded in paraffin, were assessed by gastrointestinal pathologists blinded to the experimental groups. The presence and stage of murine PanINs were analyzed as described previously [9]. For each animal approximately 100 pancreatic ducts (tail of the pancreas) were quantified and the proportion of murine PanIN-3 to the overall number of pancreatic ducts was recorded.

### Western blot analysis

Confluent cultures of PANC-1 or MiaPaCa-2 cells, grown on 35 mm tissue culture dishes, were washed twice with DMEM and incubated in serum-free medium for 4 h and then treated as described in individual experiments. The cultures were then directly lysed in 2 × SDS-PAGE sample buffer [200 mM Tris-HCl (pH 6.8), 2 mM EDTA, 0.1 M Na_3_VO_4_, 6% SDS, 10% glycerol, and 4% 2-mercaptoethanol], followed by SDS-PAGE on 4–15% gels and transfer to Immobilon-P membranes (Millipore, Billerica, MA). For detection of proteins, membranes were blocked using 5% nonfat dried milk in PBS, pH 7.2, and then incubated overnight with the desired antibodies diluted in PBS containing 0.1% Tween. Primary antibodies bound to immunoreactive bands were visualized by enhanced chemiluminescence (ECL) detection with horseradish peroxidase-conjugated anti-mouse, anti-rabbit antibody and a FUJI LAS-4000 mini luminescent image analyzer. Quantification of non saturated bands was performed using the FUJI Multi Gauge V3.0 analysis program.

### Immunofluorescence

Immunofluorescence of PANC-1 and MiaPaCa-2 cells was performed by fixing the cultures with 4% paraformaldehyde followed by permeabilization with 0.4% Triton X-100. After extensive PBS washing, fixed cells were incubated for 2h at 25°C in blocking buffer (BB), consisting of PBS supplemented with 5% bovine serum albumin and then stained at 4°C overnight with a YAP mouse mAb (1:200) diluted in BB. Subsequently, the cells were washed with PBS at 25°C and stained at 25°C for 60 min with Alexafluor 488—conjugated goat-anti mouse diluted in BB (1:100) and washed again with PBS. Nuclei were stained using a Hoechst 33342 stain (1:10,000). For staining of F-actin, fixed cells were blocked with 5% bovine serum albumin in PBS. The cells were then incubated with TRITC-conjugated phalloidin (0.25 μg/ml) in PBS for 10 min at room temperature and washed five times with PBS. Images were captured as uncompressed 24-bit TIFF files captured with an epifluorescence Zeiss Axioskop and a Zeiss (Achroplan 40/.75W objective) and a cooled (−12°C) single CCD color digital camera (Pursuit, Diagnostic Instruments) driven by SPOT version 4.7 software. AlexaFluor 488 signals were observed with a HI Q filter set 41001 and TRITC images with a HI Q filter set 41002c (Chroma Technology).

### Image analysis

For YAP localization the average fluorescence intensity in the nucleus and just outside the nucleus (cytoplasm) was measured to determine the nuclear/cytoplasmic ratios. All Image analysis was performed using Zeiss analysis imaging software. The selected cells displayed in the appropriate figures were representative of 80% of the population.

### Quantitative reverse transcription PCR (qRT-PCR)

Relative transcript expression levels of *CTGF* and *CYR61* were determined by qRT-PCR using a TaqMan Gene Expression Assay. Briefly, total RNA was extracted from cells by using a PureLink RNA Mini Kit. Reverse transcription was performed with the High-Capacity cDNA Reverse Transcription Kit using 1μg of total input RNA. The synthesized cDNA samples were used as templates for the real-time (RT) PCR analysis. All reactions were performed using the Applied Biosystems StepOne system and the amplifications were done using the TaqMan Fast Advanced Master Mix. The following primers were used; gene-specific Homo sapiens oligonucleotides for *CTGF* (Assay ID: Hs01026927_g1), *CYR61* (Assay ID: Hs99999901_s1) and gene-specific Mouse oligonucleotide primers for *Ctgf* (Assay ID: Mn00438890_m1), *Cyr61* (Assay ID: Mm00487498_m1), *Birc5* (Mn00599749_m1) the internal control was *18s* (Assay ID: Hs99999901_s1) all were from Life Technologies, Carlsbad, CA.

### Colony formation

For cell colony formation, 500 PANC-1, MiaPaCa-2, KC or KPC cells were plated into 60-mm tissue culture dishes either in DMEM containing 10% FBS or RPMI 1640 containing 5% FBS. After 24 h of incubation at 37°C, cultures were transferred to DMEM containing 3% FBS (PANC-1, MiaPaCa-2) or RPMI 1640 containing 1% FBS (KC, KPC) either in the absence or presence of statins, as indicated in the individual experiments. A colony consisted of at least 50 cells. Cell colony numbers from at least three dishes per condition were determined after 6 to 10 days of incubation.

### Transfection of GFP-AKT-PH

PANC-1 cells were transfected with the plasmid containing a cDNA encoding a green fluorescent protein (GFP) tagged-AKT pleckstrin homology domain (pcDNA3-AKT-PH-GFP was a gift from Craig Montell, Addgene plasmid # 18836) by using Lipofectamine 3000 as suggested by the manufacturer. Analysis of the cells were performed 24 h after transfection.

### Measurement of intracellular concentration of Ca^2+^ ([Ca^2+^]_i_)

PANC-1 ells grown on glass coverslips for 4–5 d were washed in Hanks’ balanced salt solution supplemented with 0.03% NaHCO3, 1.3 mM CaCl2, 0.5 mM MgCl2, 0.4 mM MgSO4, and 0.1% BSA (pH 7.4) (Hanks’ buffer). Cells were then incubated with 5 μm fura 2-tetra-acetoxy methyl ester (fura 2-AME) for 10 min in a 37 C incubator. Coverslips were then washed with Hanks’ buffer and then and mounted in a standard 1-cm path length cuvette containing Hanks’ buffer, and the cuvette was placed into a Hitachi (Tokyo, Japan) F-2000 fluorospectrophotometer. The incubation medium in the cuvette was continuously stirred at 37 C. The size of the detection window allowed measurement on the order of 10^5^ cells. Excitation was set to 340 and 380 nm, and emission signal was collected at 510 nm, all with a 10-nm bandwidth. Samples were taken every 0.5 s using associated software (F-2000 Intracellular Cation Measurement System; Hitachi Instruments). The software created the 340/380 nm ratios, which are proportional to [Ca^2+^]_i_.

### Materials

DMEM, FBS, goat anti-mouse IgG secondary antibody conjugated to Alexa Fluor 488, Fura-2 AM and all RT-qPCR reagents were obtained from Invitrogen (Carlsbad, CA). Neurotensin, insulin and phalloidin-TRITC were obtained from Sigma Chemical (St. Louis, MO). The geranylgeranyl transferase I (GGTase I) inhibitor GGTI 298, PI 3-Kinase inhibitor A66 and the MEK inhibitor PD0325901 were from R&D Systems (Minneapolis, MN). The dual PI3K/mTOR inhibitor NPV-BEZ235 was purchasedfrom Selleck Chemicals (Houston, TX). Primary antibodies used were as follows: YAP (H-9, sc-271134 and 63.1, sc-101199, final dilution 1:200 for immunofluorescence), tubulin (sc-5274; final dilution 1:400), actin (sc-47778; final dilution 1:400) and GAPDH (sc-365062; final dilution 1:400) (Santa Cruz Biotechnology); phospho-YAP Ser127 (D9W2I, 13008; final dilution 1:1000), YAP (15028; final dilution 1:1000 for western blots), phospho-p70 S6 KinaseThr389) (9205; final dilution 1:1000) and phospho-S6 Ribosomal Protein Ser240/244 (5364; final dilution 1:1000), phospho LATS Thr1079 (8654; final dilution 1:1000) and LATS2 (5888; final dilution 1:1000) were from Cell Signaling Technology (Danvers, MA). Horseradish peroxidase–conjugated anti-rabbit IgG and anti-mouse IgG were from GE Healthcare Bio-Sciences Corp (Piscataway, NJ). All other reagents were of the highest grade available.

## Results

### Statins inhibit YAP nuclear localization and activity in sparse cultures of PDAC cells

**Fig 1:**
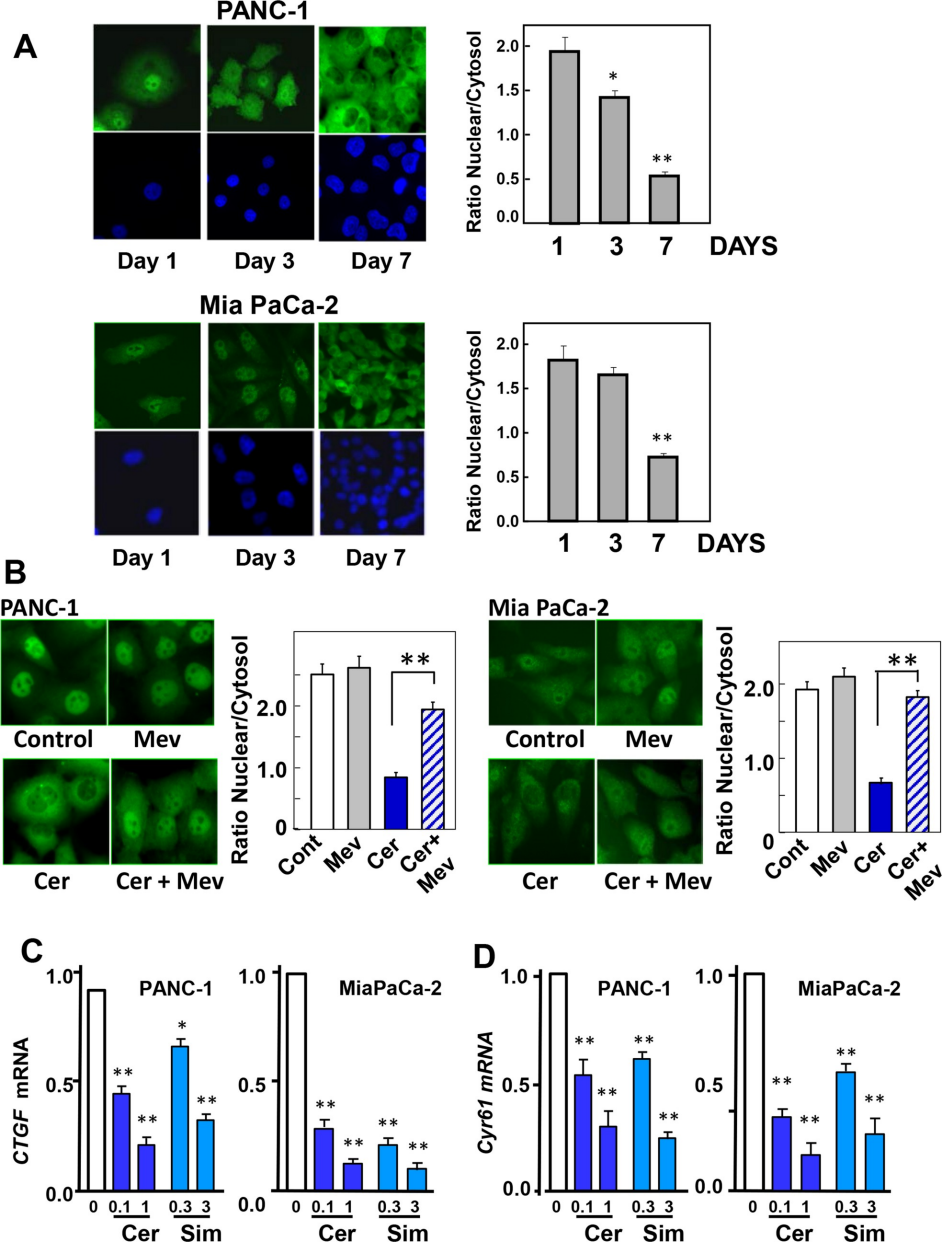
Statins induce re-distribution of YAP to the cytoplasm and inhibit YAP/TEAD-regulated genes in PDAC cells. **A**, PANC-1 and Mia PaCa-2 cells were fixed with 4% paraformaldehyde 1, 3 and 7 days after plating, as indicated. **B**, PANC-1 and Mia PaCa-2 cells were incubated in either absence or presence of 0.3 μM cerivastatin (Cer) added 1 day after plating and for 24 h. either with or without 250 μM mevalonic acid Then, the cells were fixed with 4% paraformaldehyde. In A and B the cultures were stained with an antibody that detects total YAP. Bars represent the ratio of nuclear/cytoplasm (50 to 75 cells) mean ± S.E. with similar results obtained in three independent experiments (T-test p values comparing the indicated groups to control were **p<0.01). **C** and **D**, PANC-1 and MiaPaCa-2 cells were incubated in either absence or presence of cerivastatin (Cer) at 0.1 and 1 μM or simvastatin (sim) at 0.3 and 3 μM, as indicated. Statins were added 1 day after plating and the incubation continued for 24 h. RNA was then isolated and the relative levels (n = 3) of *CTGF* (C) or *CYR61* (D) mRNA compared with 18s mRNA was measured by RT-qPCR. Data are presented as mean ± SEM of 5 independent experiments for *CTGF* and 3 independent experiments for *CYR61*. Cerivastatin and simvastatin groups vs control *p<0.05, **p<0.01.

Initially, we examined whether YAP localization depends on cell density in human PDAC cells. Specifically, PANC-1 and MiaPaCa-2 cells, plated at low densities, were fixed after 1, 3 or 7 days of culture. YAP was visualized by immunofluorescent staining. At lower cell densities (1 or 3 days in culture), YAP was localized prominently in the nucleus of PANC-1 and MiaPaCa-2 cells (**Fig 1A,** Left). In contrast, YAP was primarily in the cytoplasm of PANC-1 and MiaPaCa-2 cells maintained in culture for 7 days. The quantification of YAP nuclear/cytoplasmic ratio in multiple cells corroborated this conclusion (**Fig 1A,** Bars).

Next, we examined whether inhibition of mevalonate synthesis by statins regulates the localization of YAP in PDAC cells. Cultures of PANC-1 or MiaPaCa-2 cells were plated at low density, grown for 24 h and treated without or with cerivastatin at 0.3 μM for an additional 24 h. Then, the cultures were fixed and YAP localization was determined. As shown in **Fig 1B**, exposure to cerivastatin induced a striking re-localization of YAP from the nucleus to the cytoplasm, as verified by quantification of YAP nuclear/cytoplasmic ratio by image analysis (**Fig 1B**, Bars). Exogenously added mevalonic acid prevented the nuclear extrusion of YAP induced by exposure to cerivastatin in either PANC-1 or MiaPaCa-2 cells (**Fig 1B**). The results imply that statins induce YAP cytoplasmic localization in PDAC cells through inhibition of 3-hydroxy-methylglutaryl CoA reductase, the rate-limiting enzyme in mevalonic acid synthesis.

In line with the cytoplasmic localization induced by exposure to cerivastatin, treatment of PANC-1 or MiaPaCa-2 cells with this statin markedly reduced the mRNA levels of the YAP/TEAD-regulated gene *CTGF* (**Fig 1C**). *CTGF* is one of the best-characterized direct target gene of YAP that contains three putative YAP-TEAD binding sites (GGAATG) in its promoter region. In addition, simvastatin also inhibited *CTGF* expression in PANC-1 and MiaPaCa-2 (**Fig 1C**). Exposure to cerivastatin or simvastatin also inhibited the expression of *Cyr61*, another YAP/TEAD-regulated gene (**Fig 1D**). These results indicate that lipophilic statins regulate YAP localization and co-activator transcriptional activity in PANC-1 and MiaPaCa-2 cells.

### Statins inhibit the stimulation of YAP activity induced by neurotensin and insulin in dense cultures of PDAC cells

**Fig 2:**
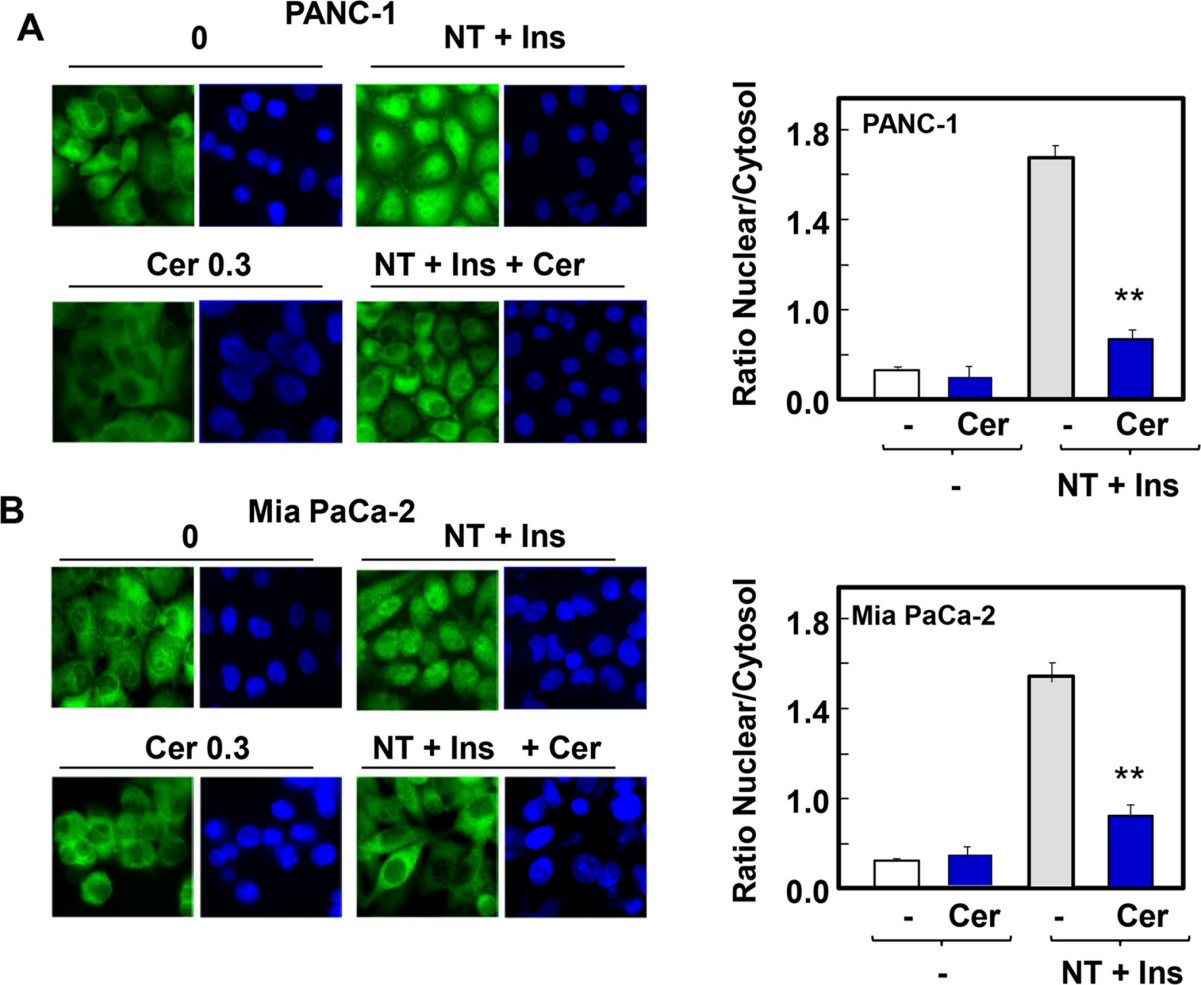
Cerivastatin inhibits YAP nuclear localization induced by stimulation with neurotensin and insulin in confluent PDAC cells. Confluent cultures of PANC-1 **(A)** or Mia PaCa-2 **(B)** cells were incubated either in the absence or presence of 0.3μM cerivastatin (Cer) for 24 h. The cultures were then stimulated without (-) or with a combination of 5 nM neurotensin and 10 ng/ml insulin (NT+Ins) for 60 min. The cultures were then washed, fixed with 4% paraformaldehyde and stained with an antibody that detects total YAP and with Hoechst 33342 to visualize the cell nuclei. Bars represent the ratio of nuclear/cytoplasm (100 to 125 cells). Cerivastatin significantly decreased NT+Ins nuclear localization of YAP as compared with untreated controls (**P < 0.01) as shown by t-test.

In previous studies, we identified potent positive crosstalk between insulin/IGF-1 receptors and G protein-coupled (GPCR) signaling systems in PDAC cells leading to early signaling and subsequent proliferation [52–57]. Recently, we reported that stimulation of dense cultures of PANC-1 or MiaPaCa-2 cells with a combination of insulin and the GPCR agonist neurotensin promoted YAP nuclear localization and stimulated the expression of YAP/TEAD-regulated genes [58]. Here, we determined whether prior exposure to statins prevents YAP nuclear localization and transcriptional activity in confluent cultures of PDAC cells challenged with these growth-promoting agonists. PDAC cells maintained in culture for 5 days were transferred to medium containing low serum and treated with or without cerivastatin at 0.3 μM for 24 h and then stimulated with a combination of 10 ng/ml insulin and 5 nM neurotensin. In line with recent results [58], stimulation of PDAC cells with insulin and neurotensin induced robust translocation of YAP to the nucleus in either PANC-1 (Fig 2A) or MiaPaCa-2 cells (Fig 2B). Prior exposure of these cells to 0.3 μM cerivastatin prevented YAP translocation to the nucleus induced by insulin and neurotensin (Fig 2), as verified by quantification of YAP nuclear/cytoplasmic ratio by image analysis (Fig 2, Bars).

**Fig 3:**
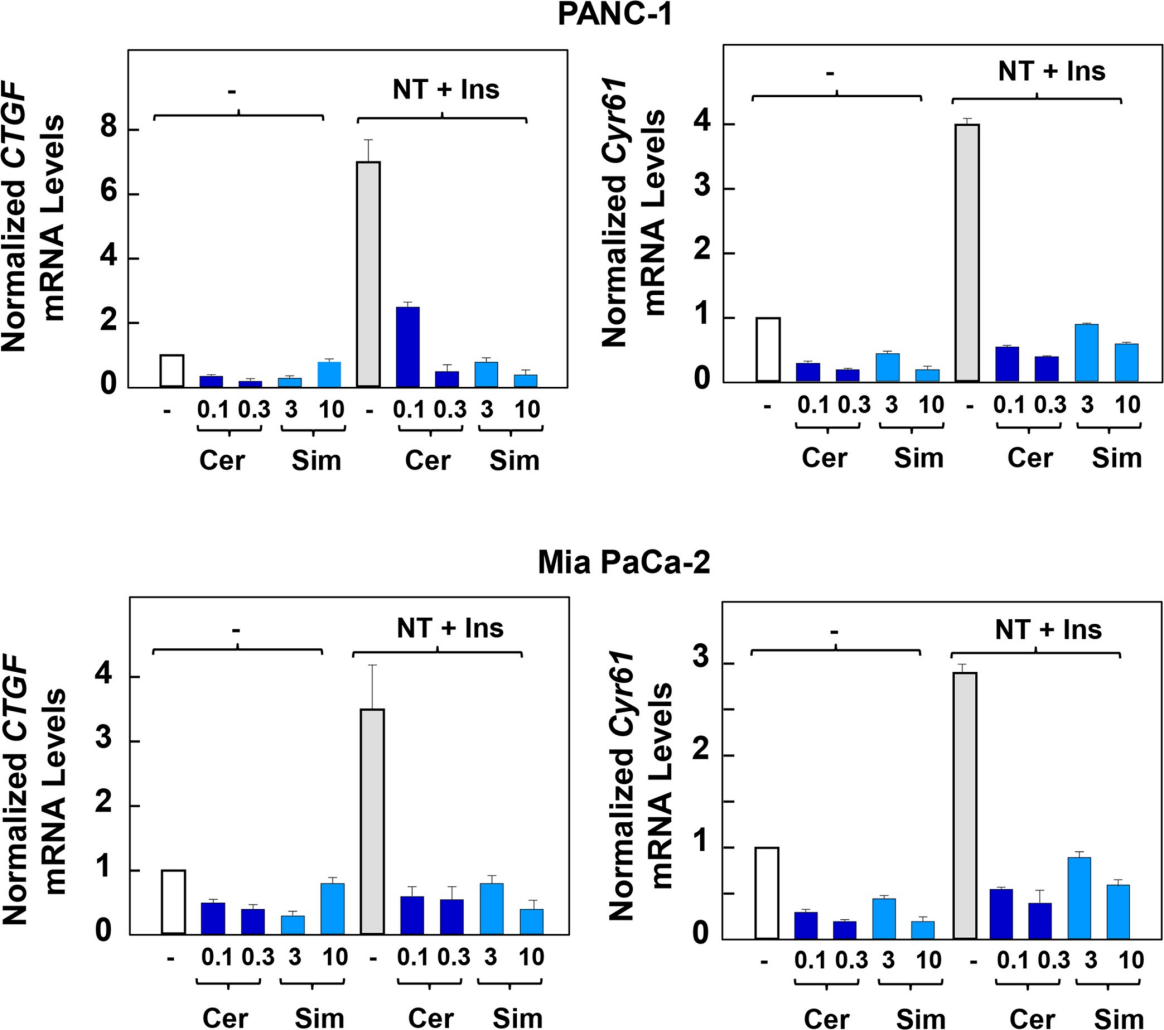
Exposure to lipophilic statins inhibits the expression of *CTGF* and *CYR61* induced by stimulation with neurotensin and insulin in PDAC cells. Confluent cultures of PANC-1 cells **(upper panel)** or MiaPaca-2 cells **(lower panel)** were incubated for 24 h either without or with cerivastatin (Cer) at 0.1 and 0.3 μM or simvastatin (sim) at 3 and 10 μM, as indicated. The cultures were then stimulated either without (-) or with a combination of 5 nM neurotensin and 10 ng/ml insulin (NT+Ins) for 60 min. RNA was isolated and the relative levels (n = 3) of CTGF or CYR61 mRNA compared with 18s mRNA was measured by RT-qPCR. Data are presented as mean ± SEM. Similar results were obtained in 3 independent experiments. Exposure to cerivastatin and simvastatin significantly decreased relative mRNA levels of CTGF and CYR61 as compared with controls (**P < 0.01) as shown by paired t-test.

Nuclear extrusion of YAP induced by statin is expected to reduce the transcriptional activity of TEAD. Consequently, we determined the effect of statins on YAP/TEAD-regulated gene expression induced by crosstalk between insulin and neurotensin. As shown in Fig 3, stimulation of confluent PANC-1 or MiaPaCa-2 cells with neurotensin and insulin induced a marked increase in the level of *CTGF* and *CYR61* transcripts, as determined by RT-qPCR. Treatment with cerivastatin completely blocked the increase in the expression of these genes at a concentration as low as 0.1 μM. Similarly, treatment with simvastatin (3 μM) prevented the increase in the mRNA levels of *CTGF* and *CYR61* induced by insulin and neurotensin. Thus, treatment with lipophilic statins induced YAP cytoplasmic localization and inhibited YAP/TEAD-regulated gene expression either in rapidly growing sparse cultures of PDAC cells (Fig 1) or in dense cultures of these cells re-stimulated via crosstalk between the GPCR agonist neurotensin and insulin (Figs 2 and 3).

### Statins inhibit colony formation by PDAC cells

Having established that statins promote cytoplasmic localization and inhibit YAP/TEAD-regulated gene expression in PDAC cells, we next determined whether statins also inhibit the proliferation of PANC-1 and MiaPaCa-2 cells by assessing the inhibitory effects of these drugs on the colony-forming ability of these PDAC cells. To this end, we plated single cell suspensions of PANC-1 or MiaPaCa-2 cells onto 60 mm dishes (500 cells per dish) in the absence or presence of increasing concentrations of different statins and determined the number of colonies formed after 8–10 days of incubation.

**Fig 4:**
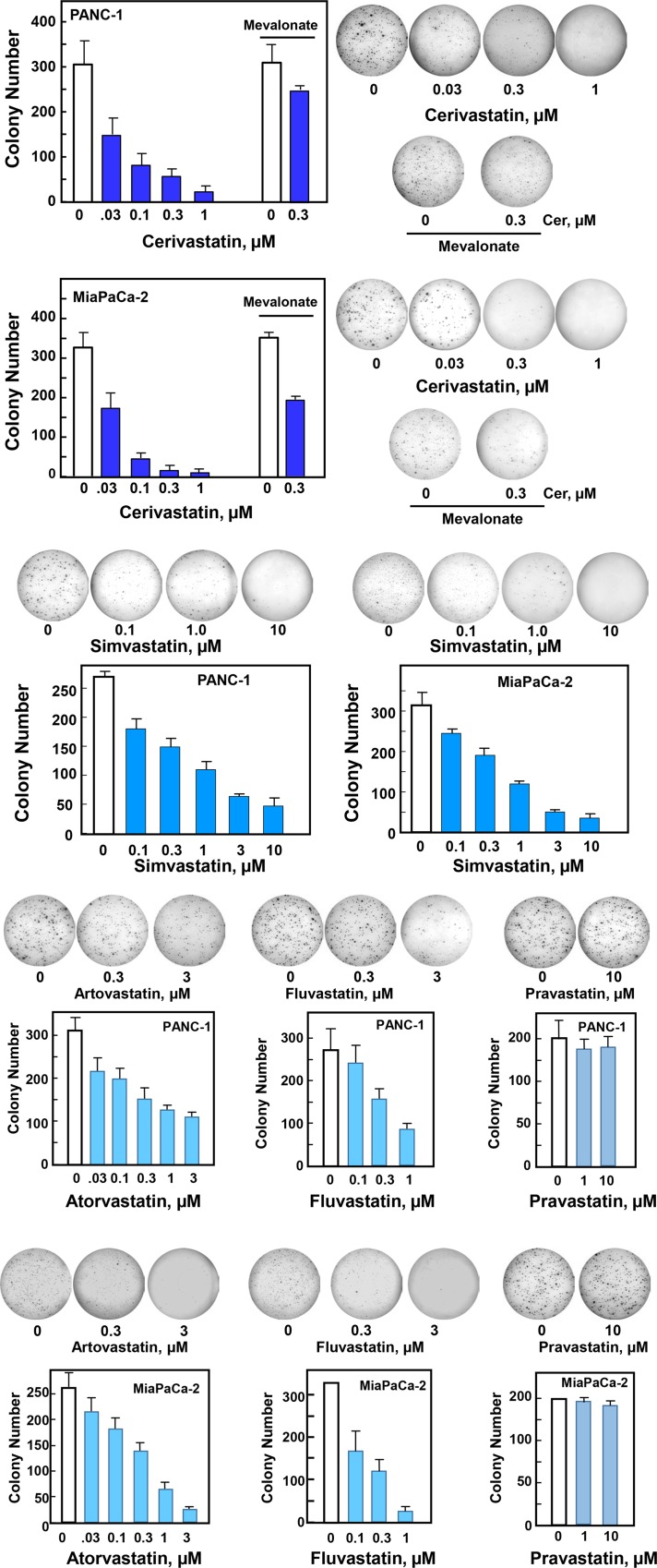
Lipophilic statins inhibit colony-formation by PDAC cells. Colony formation assays were performed as described in the Materials and Methods section. PANC-1 or MiaPaCa-2 cells were incubated for 10 days (PANC-1 cells) or 8 days (Mia PaCa-2 cells) with various concentrations of lipophilic statins, such as cerivastatin, simvastatin, atorvastatin, fluvastatin or the hydrophilic statin pravastatin, as indicated. Typical pictures are shown for selected conditions The bars represent the number of colonies (mean ± SEM; n = 4 dishes per condition). Similar results were obtained in 6 independent experiments for cerivastatin, 4 for simvastatin and 3 for atorvastatin, fluvastatin and pravastatin.

Addition of cerivastatin to the medium potently inhibited colony formation by PANC-1 or MiaPaCa-2 cells in a dose-dependent manner (Fig 4). Half-maximal inhibitory effect (IC_50_) was achieved at a concentration of cerivastatin as low as 0.03 μM. Maximal inhibition of colony formation was obtained at 0.3 μM. Addition of mevalonic acid (250 μM) reversed completely (PANC-1) or partially (MiaPaCa-2) the inhibitory effect of low concentrations (0.1–0.3 μM cerivastatin) on colony formation. These results are consistent with the notion that the statins, at low concentrations, block colony formation by PDAC cells through inhibition of 3-hydroxy-methylglutaryl CoA reductase, the rate-limiting enzyme in the generation of mevalonic acid, and with our previous results showing that knockdown of YAP/TAZ prevents colony formation by either PANC-1 or MiaPaCa-2 cells [58].

We also determined whether other lipophilic statins inhibit colony formation by PDAC cells. As shown in Fig 4, simvastatin inhibited colony formation by PANC-1 and MiaPaCa-2 cells in a dose-dependent manner with half-maximal effect achieved at 0.3 μM. In addition, atorvastatin and fluvastatin also reduced colony formation by PANC-1 cells in a dose-dependent manner. In sharp contrast, the hydrophilic statin pravastatin did not exert any inhibitory effect even at a concentration as high as 10 μM. This is likely due to the low uptake of pravastatin in pancreatic cancer cells [59]. These results indicate that cell-permeable lipophilic statins, including cerivastatin, simvastatin, atorvastatin and fluvastatin potently inhibit colony formation by PDAC cells.

### Mechanism by which statins induce cytoplasmic localization and inhibit transcriptional activity of YAP: role of YAP phosphorylation and actin organization

**Fig 5:**
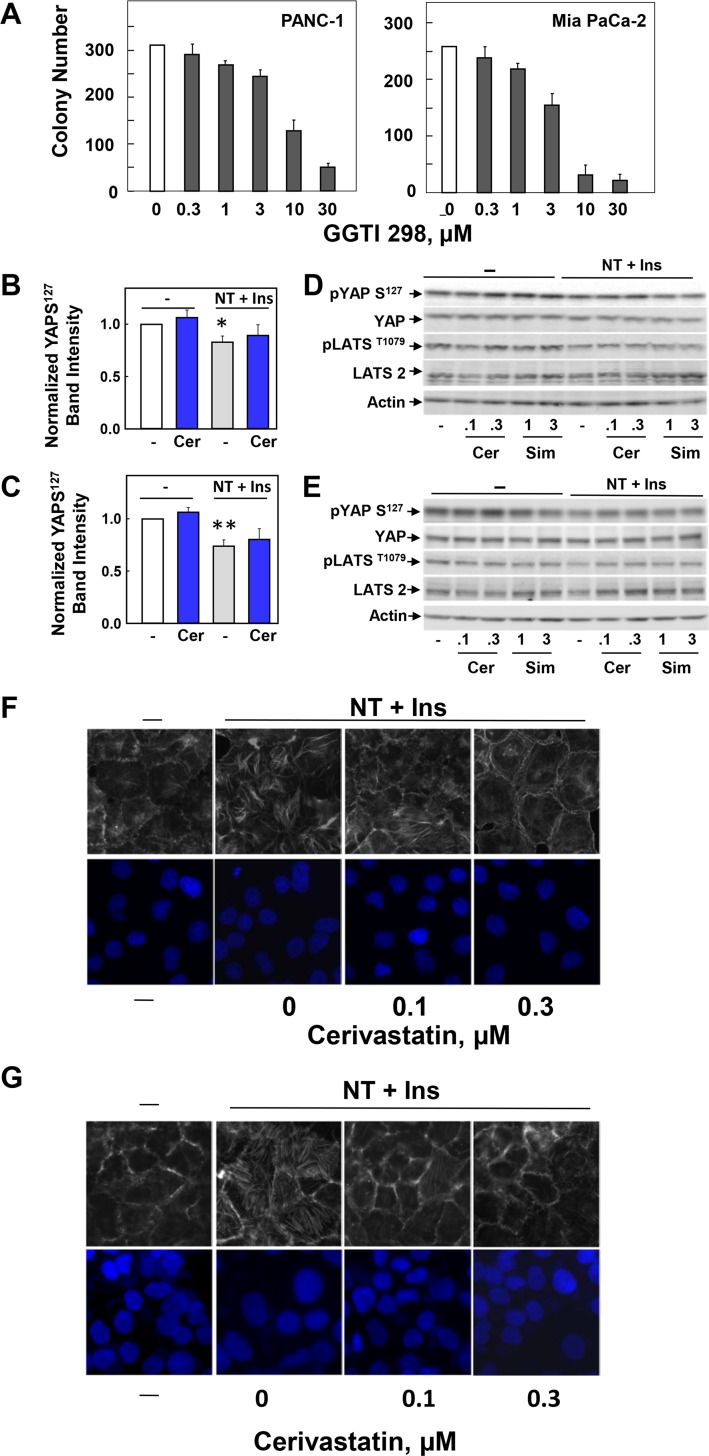
Mechanism of action of statins in PDAC cells. **A, The geranylgeranyl transferase inhibitor GGTI 298 inhibits colony formation by PDAC cells.** Colony formation assays were performed as described in the Materials and Methods section. PANC-1 or MiaPaCa-2 cells were incubated for 10 days (PANC-1 cells) or 8 days (Mia PaCa-2 cells) with or without GGTI 298 at the indicated concentrations. The bars represent the number of colonies (mean ± SEM; n = 4 dishes per condition). **B and C, Treatment with cerivastatin does not alter YAP phosphorylation at Ser**^**127**^. PANC-1 (B) and Mia PaCa-2 (C) cells were treated for 24 h either in the absence or presence of cerivastatin (Cer, 0.3 μM) for 24h. The cultures were then stimulated with 5 nM neurotensin and 10 ng/ml insulin (NT+Ins) for 30 min as indicated, and lysed with SDS–PAGE sample buffer. The samples were analyzed by SDS-PAGE and immunoblotting with YAP Ser^127^ and YAP antibodies. Quantification of phosphorylated YAP Ser^127^ was performed using Multi Gauge V3.0. from 9 independent experiments, T-test p values comparing the indicated groups to control were * p< 0.05; **p < .0.01. **D and E, Exposure to either cerivastatin or simvastatin does not alter YAP phosphorylation at Ser**^**127**^. PANC-1 and Mia PaCa-2 cells were treated for 24 h either in the absence or presence of cerivastatin (Cer) or simvastatin (Sim) at the indicated concentrations (in μM) for 24h. The cultures were then stimulated with 5 nM neurotensin and 10 ng/ml insulin (NT+Ins) for 30 min as indicated, and lysed with SDS–PAGE sample buffer. The samples were analyzed by SDS-PAGE and immunoblotting with antibodies that detect phospho YAP Ser^127^, YAP, phospho LATS Thr^1079^ and LATS2 antibodies. Equal loading was verified by immunoblotting with actin antibody. Similar results were obtained in 4 independent experiments. **F and G, Cerivastatin inhibits the assembly of actin stress fibers induced by stimulation with insulin and neurotensin.** PANC-1 (F) and Mia PaCa-2 cells (G) were treated for 24 h either in the absence or presence of cerivastatin at the indicated concentrations for 24h prior to stimulation with 5 nM neurotensin and 10 ng/ml insulin (NT+Ins) for 30 min. The cultures were then washed, fixed with 4% paraformaldehyde, and stained with TRITC-conjugated phalloidin and Hoechst 33342. Similar results were obtained in 3 independent experiments.

It is widely recognized that statins deplete several important lipid intermediates, including geranylgeranyl pyrophosphate (GGPP), a critical isoprenoid in Rho prenylation. A key enzyme in this process is geranylgeranyl transferase (GGTase) that catalyzes the transfer of GG to Rho. In line with this notion, the selective inhibitor of GGTase, GGTI 298, mimicked the inhibitory effect of statins on colony formation by PDAC cells (Fig 5A).

The precise mechanism linking Rho to YAP activity in PDAC cells remains incompletely understood. In other cell types, statin treatment appears to increase YAP phosphorylation at Ser^127^ through a Hippo-independent but Rho-inhibited kinase pathway [35, 37]. So far, the putative Rho-sensitive kinase remains unidentified. In contrast, we found that exposure to cerivastatin did not alter the phosphorylation of YAP at Ser^127^ in either PANC-1 (Fig 5B) or MiaPaCa-2 (Fig 5C) cells incubated either without or with neurotensin and insulin. These results were reproduced in 9 separate experiments. In addition, we did not detect any consistent increase in the phosphorylation of YAP at Ser^127^ or change in total YAP in PANC-1 (Fig 5D) or MiaPaCa-2 (Fig 5E) cells treated with different concentrations of cerivastatin or simvastatin without or with stimulation by insulin and neurotensin. Accordingly, we did not detect any change in LATS phosphorylation at Thr^1079^ or in total LATS in response to these treatments in PANC-1 (Fig 5D) or MiaPaCa-2 (Fig 5E) cells These results indicate that statins inhibit YAP nuclear localization and co-activator activity independently of its phosphorylation at Ser^127^ in PDAC cells.

The organization of the actin cytoskeleton plays a central role in the regulation of YAP localization and transcriptional activity independently of YAP phosphorylation [13]. Statins disrupt Rho-dependent actin remodeling in at least some cell types [37] but their effect on the actin organization of PDAC cells has not been determined. Consequently, we examined the effect of statin exposure on actin organization in PDAC cells challenged with insulin and neurotensin, as visualized by phalloidin staining. Stimulation of PDAC cells with the combination of insulin and neurotensin induced a marked increase in the assembly of actin stress fibers, a surrogate marker of Rho signaling. Prior exposure to cerivastatin (0.1–0.3 μM) blunted stress fiber formation in PANC-1 (Fig 5F) and MiaPaCa-2 (Fig 5G) cells. These results imply that statins inhibit YAP via a Hippo pathway-independent disruption of the actin cytoskeleton organization.

In order to test further the specificity of the statins in PDAC cells, we examined whether treatment with cerivastatin inhibts other cellular responses induced by crosstalk between insulin and neurotensin. Because statins inhibited PI3K activity in other cell types [60], and this enzyme contributes to YAP activation in PDAC cells [58], we determined the effect of cerivastatin on the activity of PI3K in intact PANC-1 cells. To ascertain whether treatment with cerivastatin diminishes the accumulation of phosphatidylinositol (3,4,5)-trisphosphate (PIP3) in the plasma membrane, we monitored the redistribution of Akt-pleckstrin homology domain-green fluorescent protein (Akt-PH-GFP) in single PANC-1 cells. In line with our previous results, stimulation with insulin and neurotensin markedly increased membrane accumulation of Akt-PH-GFP [58]. The translocation of the PIP3 sensor to the plasma membrane was not prevented by prior exposure to cerivastatin at a concentration as high as 1 μM (S1 Fig). In contrast, the class I p110α specific inhibitor A66, tested as a positive control, abolished the movement of Akt-PH-GFP to the plasma membrane (S1 Fig). In line with these results, treatment with cerivastatin did not prevent the stimulation of p70S6K phosphorylated on Thr^389^, a direct marker of mTORC1 activity, and the phosphorylation of p70S6K substrate, S6 on Ser^240^ induced by stimulation with neurotensin and insulin (S1 Fig). As a control, we verified that treatment with BEZ-235, a dual PI3K/mTOR inhibitor, completely blocked the phosphorylation of these proteins, as expected [61].

Given that an increase in the intracellular concentration of Ca^2+^ ([Ca^2+^]_i_) is one of the earliest events induced by GPCR agonists via heterotrimeric G proteins rather than monomeric GTPases (such as Rho), we also determined the effect of statins on the increase in [Ca^2+^]_i_. Prior exposure to cerivastatin did not prevent the increase in [Ca^2+^]_i_ induced by subsequent stimulation with neurotensin in PANC-1 cells (S1 Fig). Collectively, these results indicate that statins inhibit YAP activation through disruption of actin organization without increasing YAP phosphorylation at Ser^127^ or interfering with other signaling events in PDAC cells, including PI3K/mTORC1 activation, and Ca^2+^ mobilization.

To further define the role of Rho, YAP and LATS in human PDAC, we analyzed the association of their expression to PDAC survival in human PDAC. We used the Pathology Atlas [62] based on the integration of publicly available data from The Cancer Genome Atlas (TCGA) and data generated within the framework of the Human Protein Atlas (HPA) and analyzed transcriptomics and survival in 176 PDAC patients [62]. In agreement with previous findings, increased YAP expression is strongly associated (p<0.001) with unfavorable prognosis [17]. Similarly, expression of *RhoA* and *RhoC* mRNA levels is associated with poor survival of PDAC (S2 Fig). In contrast, increased mRNA expression of *LATS1*/*2*, which phosphorylate YAP at Ser^127^, is not associated with PDAC survival (S2 Fig). These findings support the notion that the Rho GTPases promote YAP activity and poor PDAC survival and thus, are in line with the conclusions drawn from the mechanistic studies presented in Fig 5.

### Effect of statins on YAP localization and activity in murine KC and KPC cells

**Fig 6:**
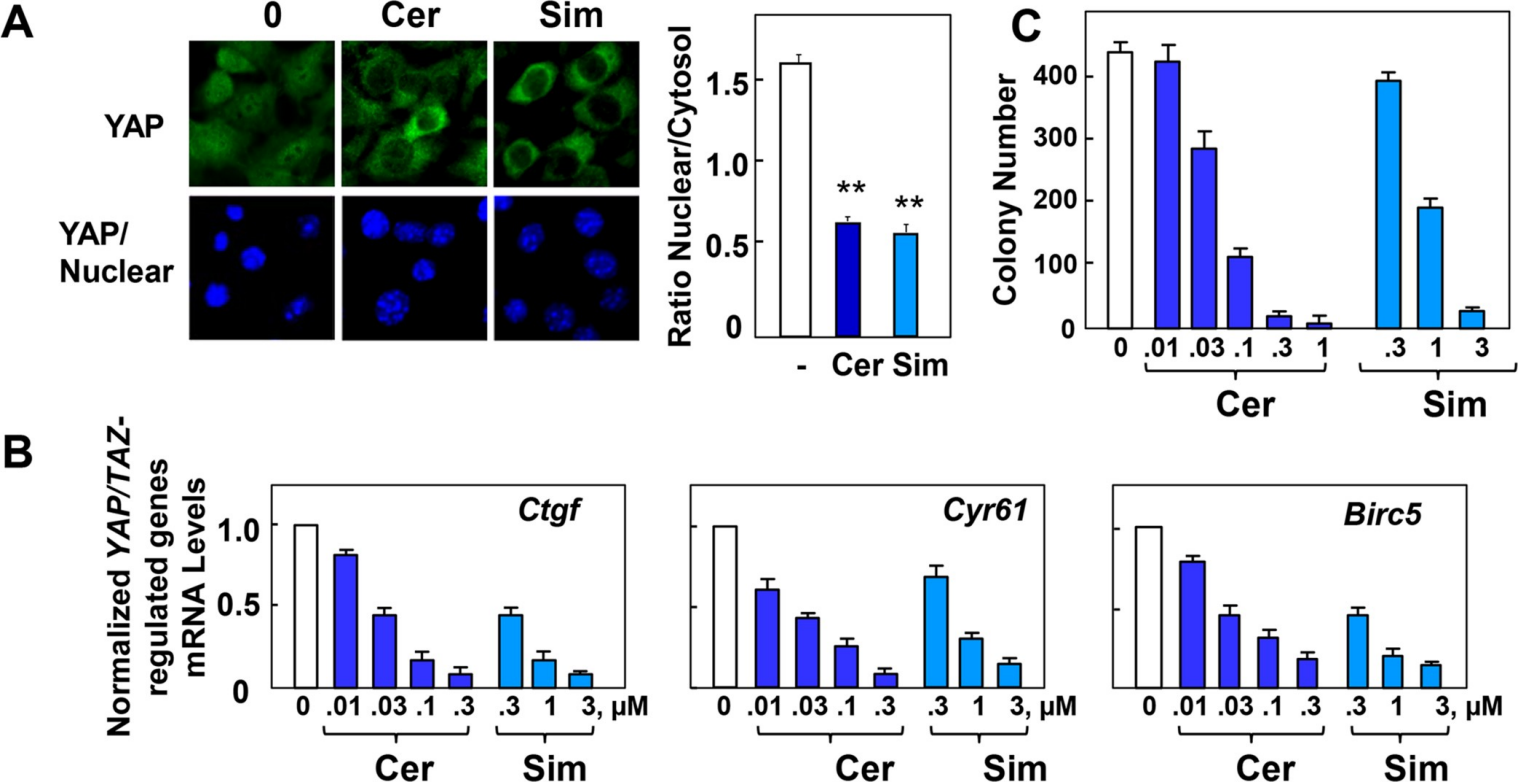
Statins inhibit YAP nuclear localization, colony formation and the expression of *Ctgf*, *Cyr61* and *Birc5* in KC cells. **A,** KC cells 1 day after plating were incubated either in absence or presence of 0.3 μM cerivastatin (Cer) or 3 μM simvastatin (Ser) for 24 h. Then, the cells were then fixed with 4% paraformaldehyde and stained with an antibody that detects total YAP and with Hoechst 33342 to visualize the cell nuclei. Bars represent the ratio of nuclear/cytoplasm (50 to 75 cells). **P 0.01 as shown by paired t-test. **B,** KC cells were incubated either in absence or presence of cerivastatin (Cer) or simvastatin (Sim) at the indicated concentrations. Statins were added 1 day after plating and the incubation continued for 24 h. RNA was then isolated and the relative levels (n = 3) of *Ctgf*, *Cyr61* and *Birc5* mRNA compared with 18s mRNA were measured by RT-qPCR. Data are presented as mean ± SEM. Similar results were obtained in 3 independent experiments. **C,** KC cells were incubated for 6 days with various concentrations of cerivastatin or simvastatin, as indicated. The bars represent the number of colonies (mean ± SEM; n = 4 cultures per condition).

Next, we examined the effects of cerivastatin and simvastatin on YAP localization, YAP/TEAD-regulated gene expression and colony formation in KC cells. Treatment of KC cells with either cerivastatin or simvastatin induced striking YAP translocation from the nucleus to the cytoplasm, as verified by quantification of YAP nuclear/cytoplasmic (Fig 6A). Exposure to cerivastatin or simvastatin also inhibited the expression of the YAP/TEAD-regulated genes *Ctgf*, *Cyr61* and *Birc5* (Fig 6B). Furthermore, addition of either cerivastatin or simvastatin strikingly inhibited colony formation by KC cells in a dose-dependent manner (Fig 6C).

A recent study concluded that statin-induced depletion of mevalonic acid destabilizes mutant p53 protein and thereby exerts a more potent growth-inhibitory effects in cancer cells harboring p53 mutant [63]. Although this mechanism could explain, at least partly, the statin sensitivity of the human pancreatic cancer cells used in this study (PANC-1 and MiaPaCa-2) which contain mutated *TP53*, the KC cells express wild type p53. In order to determine the contribution of p53 mutation to statin sensitivity in murine PDAC cells, we also determined the effects of statins on colony formation and YAP/TEAD-regulated genes in cells isolated from the pancreas of mice expressing both KrasG12D and mutant p53 (KPC mice). We found that cerivastatin and simvastatin inhibited colony formation and expression of *Ctgf* and *Cyr61* in KPC cells at concentrations similar to those that produced inhibitory effects in KC cells (S3 Fig). The results indicate that the inhibitory effects of statins do not depend on the expression of mutant p53 in murine PDAC cells.

### Effect of simvastatin on initial stages of PDAC development in vivo

**Fig 7:**
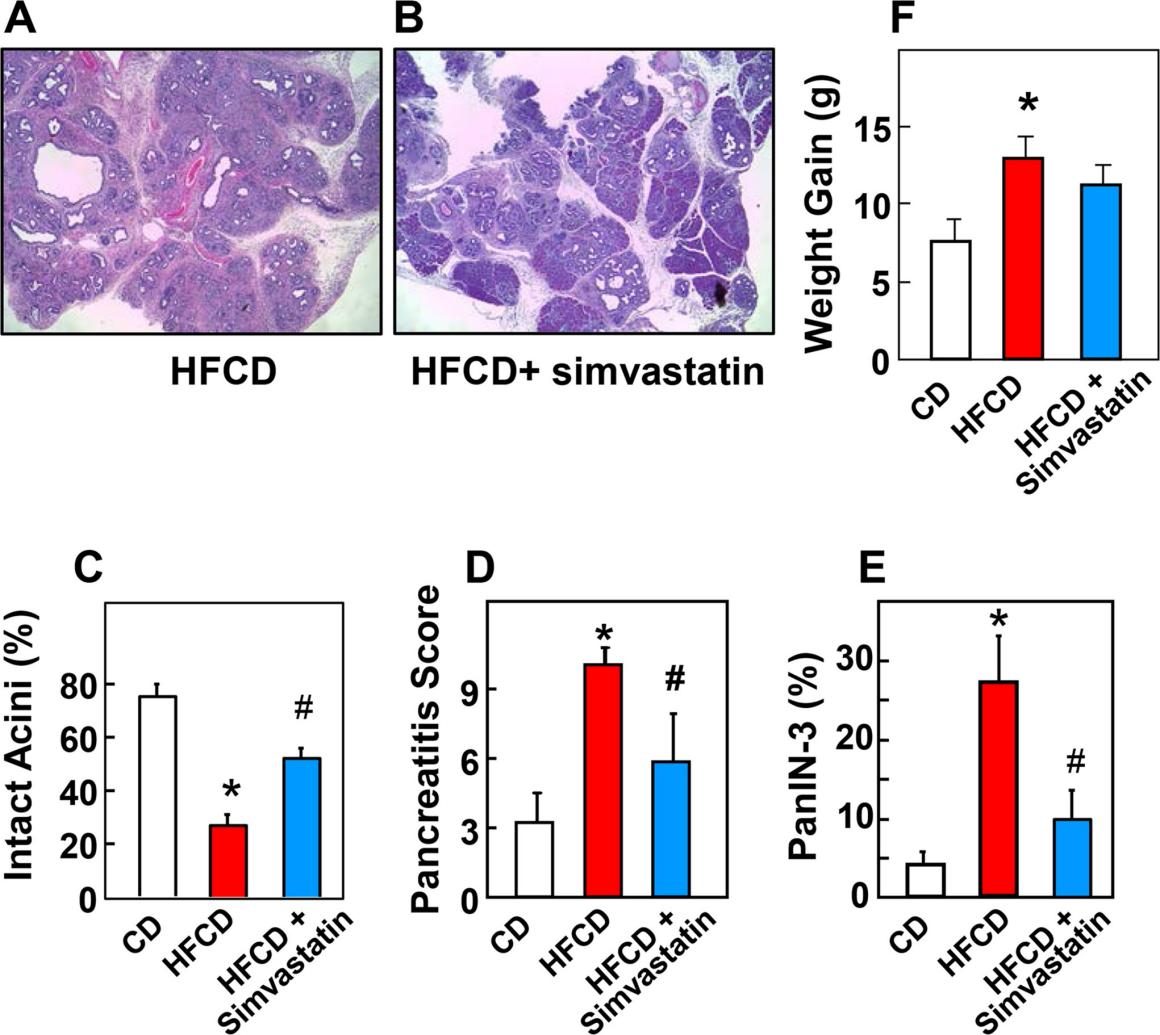
Simvastatin prevents the disruption in histologic pancreatic architecture induced by DIO in KC mice. **A and B,** Representative H&E staining of the pancreas of a KC mouse fed the HFCD (A) or HFCD plus simvastatin (B). **C,** Percentage (%) of intact acini in KC mice fed the CD, HFCD or HFCD plus simvastatin at 3 months. **D,** Pancreatitis score in KC mice fed CD, HFCD or HFCD plus simvastatin at 3 months. **E,** Percentage (%) of PanIN-3 lesions in KC mice fed CD, HFCD or HFCD plus simvastatin at 3 months. **F,** Simvastatin has no effect on weight gain in KC mice fed with either CD, HFCD, or HFCD plus simvastatin at 3 months. The number of mice used were: CD = 10; HFCD = 14; HFCD+simvastatin = 14. Values are means ± s.d. *p < 0.05 vs. CD, #p < 0.05 vs. HFCD.

Recent studies demonstrated that the YAP/TAZ pathway plays a critical role in promoting the initial stages of PDAC development, namely depletion of intact acini (acinar-ductal metaplasia) and formation of PanIN lesions [14, 15]. Consequently, we determined the effect of statin on pancreatic pre-neoplastic lesions in KC mice subjected to diet-induced obesity that accelerates the appearance and stage of these lesions [16]. Specifically, cohorts of KC mice were fed CD, HFCD, or HFCD plus simvastatin (40mg/kg orally) for 3 months. In line with our previous results [8, 9], administration of the obesogenic diet induced a marked increase in acini depletion, inflammation and in PanIN-3 formation. Treatment with simvastatin significantly attenuated the loss of intact acini (histological images in Fig 7A and 7B; quantification in Fig 7C), markedly reduced the pancreatitis score (calculated as in [8, 9]) and the formation of PanIN-3 lesions (quantification in Fig 7E) induced by DIO. At the dose used, simvastatin did not produce any adverse clinical effects, and had no effect on weight gain (Fig 7E).

## Discussion

PDAC is a clinically aggressive disease that is anticipated to become the 2^nd^ cause of cancer fatalities during the next decade [2]. As current therapeutic approaches are limited, novel targets and agents for chemoprevention are urgently needed and will most likely arise from a more detailed understanding of the signaling mechanisms that stimulate the promotion and progression of pre-malignant (initiated) cells into invasive pancreatic cancer cells.

The highly conserved transcriptional co-activator YAP and its paralog TAZ, originally identified in *Drosophila*, are emerging as potent oncogenes for a variety of cell types [20, 64]. In this context, a number of studies indicate that YAP and TAZ are overactive in PDAC patient tumor samples [24, 65, 66] and associated with poor survival [17]. The YAP/TAZ pathway assumes an added importance in PDAC because YAP is a key downstream target of Ras signaling required for PanIN progression into invasive PDAC [14, 15]. Furthermore, YAP not only acts downstream of K-ras but its hyper-activation can bypass the need of oncogenic K-ras in PDAC development [7, 24] and plays a critical role in the crosstalk between GPCR agonists and insulin [58]. In the light of these new developments, there is intense interest in targeting YAP/TAZ in PDAC, given the urgent need of novel strategies for combating this lethal disease.

Given the critical role of gene-regulatory programs in cancer development, inhibition of transcription factors or their co-activators in cancer cells is a logical but challenging strategy. Using sparse cultures of these PDAC cells, we show here that exposure to lipophilic statins induces robust translocation of YAP from the nucleus to the cytoplasm. Using dense cultures of PDAC cells, we demonstrate that statins block the translocation of YAP to the nucleus induced by crosstalk between insulin and neurotensin signaling systems, a potent mitogenic combination for these cells. Collectively, these results demonstrate that treatment with statins induces YAP cytoplasmic sequestration in PDAC cells, in agreement with recent findings in other cancer cell types [35–37].

When YAP/TAZ localize to the nucleus, they bind and activate primarily the TEA-domain DNA-binding transcription factors (TEAD 1–4) thereby stimulating the expression of a variety of genes [67], including *CTGF* and *CYR61*, well established YAP/TEAD-regulated genes. Here, we found that lipophilic statins, including cerivastatin and simvastatin, markedly reduced the mRNA levels of *CTGF* and *CYR61* in sparse cultures of PANC-1 and MiaPaCa-2 cells and blocked the increase in the expression of these genes induced by stimulation with neurotensin and insulin in dense and serum-deprived cultures. Taken together, the results of YAP localization and expression of YAP/TEAD-regulated genes indicate that statins block YAP activity by restricting its localization to the cytoplasm. In agreement with the notion that YAP nuclear activity stimulates the expression of genes, including *CTGF* and *CYR61*, implicated in PDAC cell proliferation, we found that cell-permeable lipophilic statins, including cerivastatin, simvastatin, atorvastatin and fluvastatin potently inhibit colony formation by PANC-1 and MiaPaCa-2 cells whereas the hydrophilic statin pravastatin did not have any detectable inhibitory effect even at high concentrations.

We next examined the mechanism by which statins regulate YAP localization in PDAC cells. Canonical Hippo signals are transduced through a serine/threonine kinase cascade wherein Mst1/2 kinases, in complex with Sav1, phosphorylate and activate LATS1/2, in complex with its regulatory protein MOB1/2 [12]. The activated LATS1/2 catalyzes the phosphorylation of YAP at five different sites, including Ser^127^, a residue proposed to mediate cytoplasmic retention in its phosphorylated state, though the phosphorylation of this residue did not impair nuclear import of YAP in a variety of cell types [13]. Previous studies resulted in differing conclusions concerning the role of the Hippo pathway and YAP phosphorylation in the mechanism by which statins promote YAP localization to the cytoplasm [35, 37] but none of these studies were performed in PDAC cells. Our results demonstrate that exposure to lipophilic statins, at concentrations that markedly inhibit YAP nuclear localization, YAP/TEAD-regulated gene expression and colony formation, did not induce any significant change in the phosphorylation of YAP at Ser^127^ or LATS at Thr^1079^ in PDAC cells. Accordingly, we conclude that statins inhibit YAP activity through a Hippo pathway-independent mechanism.

A considerable body of evidence indicates that multiple signaling inputs regulate the activity of YAP through the organization of filamentous (F) actin [68, 69]. Rho plays a critical role in F-actin organization and in the assembly of actin stress fibers in a variety of cell types [70]. Interestingly, Rho-induced actomyosin-based tension causes YAP/TAZ activation, as well as proposed to “open” nuclear pores for YAP/TAZ nuclear import [71, 72]. Statins are specific inhibitors of the 3-hydroxy-methylglutaryl (HMG) CoA reductase [32], the rate-limiting enzyme in the generation of mevalonate, the first step in the biosynthesis of isoprenoids, leading to farnesyl pyrophosphate (FPP), geranylgeranyl pyrophosphate (GG-PP) and cholesterol. The transfer of the geranylgeranyl moiety to a COOH-terminal cysteine of Rho GTPases is critical for their function. Here, we produced several lines of evidence indicating that statins inhibit YAP through a Rho-mediated mechanism: 1) exogenously added mevalonate reversed the inhibitory effect of low concentrations of statins on nuclear localization and at least in part, on colony formation of PDAC cells; 2) a selective inhibitor of GGTase, GGTI 298, mimicked the inhibitory effect of statins on colony formation by PDAC cells; 3) treatment of PDAC cells with cerivastatin drastically reduced stress fiber formation. These results, together with the dissociation from YAP phosphorylation discussed above, imply that statins inhibit YAP activity by interfering with the polymerization of the actin cytoskeleton. In line with these mechanistic studies, we found that the increased expression of Rho GTPases (RhoA, RhoC) is associated with poor survival in PDAC patients.

We extended these findings to murine PDAC and to a mouse model of acinar-ductal metaplasia, PanIN formation and PDAC development. Initially, we demonstrated that treatment of cells isolated from the pancreas of KC or KPC mice with lipophilic statins induced striking YAP translocation from the nucleus to the cytoplasm, inhibited the expression of the YAP/TEAD-regulated genes *Ctgf*, *Cyr61* and *Birc5* and profoundly inhibited colony formation by these cells. The results with murine cells are in agreement with the findings in human PDAC cells and additionally imply that mutation of p53 is not necessary for high statin sensitivity, at least in murine cells.

Subsequently, we determined the impact of a lipophilic statin on early neoplastic changes in the pancreas of KC mice subjected to DIO, a condition that markedly accelerates the development of pancreatic neoplastic lesions. We found that treatment with simvastatin significantly reversed the loss of intact acini induced by DIO and markedly reduced the formation of PanIN-3 lesions. Collectively, our results provide robust preclinical evidence that statin administration provides a plausible strategy to be considered in the prevention and treatment of PDAC.

In line with this conclusion, recent epidemiological studies imply that the use of statins is associated with beneficial effects in PDAC [38–47], and other malignancies [48]. Although a recent study failed to detect an effect of statins in lowering PDAC risk [73], a follow up study of the same data reported an increased survival in PDAC patients with regular pre-diagnosis use of statins [74]. Taken together with the findings presented here and the lack of efficient strategies to oppose PDAC development, these studies warrant a comprehensive evaluation of the lipophilic statins in the primary or secondary chemoprevention of this devastating disease.

## Supporting information

S1 Fig Treatment with cerivastatin does not prevent PI3K activation or increase in [Ca^2+^]_i_ in PANC-1 cells. **A**: PANC-1 cells were transiently transfected with a plasmid encoding a fusion protein between GFP and the PH domain of AKT (AKT-PH-GFP). After 24h, the cultures were incubated in DMEM without or with cerivastatin at the indicated concentrations for 18h prior to stimulation with 5 nM neurotensin and 10 ng/ml insulin. Other cultures weretreated with the class I p110α specific inhibitor A66 at 10 μM, tested as a positive control The intracellular distribution of AKT-PH-GFP was monitored under a fluorescence microscope. The selected cells displayed in the figures were representative of 90% of the population of GFP-positive cells. **B**: PANC-1 cells were treated for 24 h either in the absence or presence of cerivastatin (Cer) at the indicated concentrations for 24h. Other cultures were incubated for 2h with the either the MEK inhibitor PD0325901 (1μM, PD) or the dual PI3K/mTOR inhibitor NPV-BEZ235 (1μM, BEZ). All cultures were then stimulated with 5 nM neurotensin and 10 ng/ml insulin (NT+Ins) for 30 min as indicated, and lysed with SDS–PAGE sample buffer. The samples were analyzed by SDS-PAGE and immunoblotting with phospho-p70 S6 KinaseThr-389 and phospho-S6 Ribosomal Protein Ser-240/244. Equal loading was verified by immunoblotting with GAPDH antibody.Similar results were obtained in 2 independent experiments. **C:** PANC-1 cells were incubated without or with cerivastatin at the indicated concentrations for 18h prior to stimulation with 5 nM neurotensin. Intracellular [Ca^2+^]_i_ was monitored as described in Materials and Methods.
(TIF)

S2 Fig Kaplan-Meier plots for RHO and LATS expression in PDAC. Images were reproduced from the Human Protein Atlas (version 17) available from www.proteinatlas.org The link is: http://www.proteinatlas.org/ENSG00000137693YAP1/pathology/tissue/pancreatic+cancerS1
(TIF)

S3 Fig Statins inhibit colony formation and the expression of CTGF, CYR61 and BIRC5 in KPC cells. **A**, KPC cells were incubated for 6 days with various concentrations of cerivastatin or simvastatin, as indicated. The bars represent the number of colonies (mean ± SEM; n = 4 dishes per condition). **B**, KPC cells were incubated either in absence or presence of cerivastatin (Cer) or simvastatin (Sim) at the indicated concentrations. Statins were added 1 day after plating and the incubation continued for 24 h. RNA was then isolated and the relative levels (n = 3) of CTGF, CYR61 and BIRC5 mRNA compared with 18s mRNA were measured by RT-qPCR. Data are presented as mean ± SEM. Similar results were obtained in 3 independent experiments.
(TIF)

## References

[1] Siegel RL, Miller KD, Jemal A. Cancer statistics, 2018. CA Cancer J Clin. 2018;68(1):7–30. 10.3322/caac.21442 29313949

[2] Rahib L, Smith BD, Aizenberg R, Rosenzweig AB, Fleshman JM, Matrisian LM. Projecting Cancer Incidence and Deaths to 2030: The Unexpected Burden of Thyroid, Liver, and Pancreas Cancers in the United States. Cancer Res. 2014;74(11):2913–21. 10.1158/0008-5472.CAN-14-0155 24840647

[3] Hingorani SR, Petricoin EF, Maitra A, Rajapakse V, King C, Jacobetz MA, et al Preinvasive and invasive ductal pancreatic cancer and its early detection in the mouse. Cancer Cell. 2003;4(6):437–50. . 14706336 10.1016/s1535-6108(03)00309-x

[4] Maitra A, Fukushima N, Takaori K, Hruban RH. Precursors to invasive pancreatic cancer. Adv Anat Pathol. 2005;12(2):81–91. . 15731576 10.1097/01.pap.0000155055.14238.25

[5] Jones S, Zhang X, Parsons DW, Lin JC, Leary RJ, Angenendt P, et al Core signaling pathways in human pancreatic cancers revealed by global genomic analyses. Science. 2008;321(5897):1801–6. 10.1126/science.1164368 . 18772397 PMC2848990

[6] Biankin AV, Waddell N, Kassahn KS, Gingras M-C, Muthuswamy LB, Johns AL, et al Pancreatic cancer genomes reveal aberrations in axon guidance pathway genes. Nature. 2012;491(7424):399–405. 10.1038/nature11547 PMC3530898. 23103869 PMC3530898

[7] Ying H, Dey P, Yao W, Kimmelman AC, Draetta GF, Maitra A, et al Genetics and biology of pancreatic ductal adenocarcinoma. Genes Dev. 2016;30(4):355–85. 10.1101/gad.275776.115 26883357 PMC4762423

[8] Dawson DW, Hertzer K, Moro A, Donald G, Chang HH, Go VL, et al High Fat, High Calorie Diet Promotes Early Pancreatic Neoplasia in the Conditional KrasG12D Mouse Model. Cancer Prev Res (Phila). 2013;6:1064–73. 10.1158/1940-6207.CAPR-13-0065 . 23943783 PMC3835151

[9] Chang H-H, Moro A, Takakura K, Su H-Y, Mo A, Nakanishi M, et al Incidence of pancreatic cancer is dramatically increased by a high fat, high calorie diet in KrasG12D mice. PLOS ONE. 2017;12(9):e0184455 10.1371/journal.pone.0184455 28886117 PMC5590955

[10] Albini A, DeCensi A, Cavalli F, Costa A. Cancer Prevention and Interception: A New Era for Chemopreventive Approaches. Clin Cancer Res. 2016;22(17):4322–7. 10.1158/1078-0432.CCR-16-0695 27220959

[11] Avruch J, Zhou D, Fitamant J, Bardeesy N, Mou F, Barrufet LR. Protein kinases of the Hippo pathway: Regulation and substrates. Semin Cell Dev Biol. 2012;23(7):770–84. 10.1016/j.semcdb.2012.07.002 22898666 PMC3489012

[12] Meng Z, Moroishi T, Guan K-L. Mechanisms of Hippo pathway regulation. Genes Dev. 2016;30(1):1–17. 10.1101/gad.274027.115 26728553 PMC4701972

[13] Totaro A, Panciera T, Piccolo S. YAP/TAZ upstream signals and downstream responses. Nat Cell Biol. 2018;20(8):888–99. 10.1038/s41556-018-0142-z 30050119 PMC6186418

[14] Zhang W, Nandakumar N, Shi Y, Manzano M, Smith A, Graham G, et al Downstream of Mutant KRAS, the Transcription Regulator YAP Is Essential for Neoplastic Progression to Pancreatic Ductal Adenocarcinoma. Sci Signal. 2014;7(324):ra42–ra. 10.1126/scisignal.2005049 24803537 PMC4175524

[15] Gruber R, Panayiotou R, Nye E, Spencer-Dene B, Stamp G, Behrens A. YAP1 and TAZ Control Pancreatic Cancer Initiation in Mice by Direct Up-regulation of JAK–STAT3 Signaling. Gastroenterology. 2016;151(3):526–39. 10.1053/j.gastro.2016.05.006 27215660 PMC5007286

[16] Eibl G, Rozengurt E. KRAS, YAP, and obesity in pancreatic cancer: A signaling network with multiple loops. Semin Cancer Biol. 2017 10.1016/j.semcancer.2017.10.007 PMC5916582 29079305

[17] Rozengurt E, Sinnett-Smith J, Eibl G. Yes-associated protein (YAP) in pancreatic cancer: at the epicenter of a targetable signaling network associated with patient survival. Signal Transduct Targeted Ther. 2018;3(1):11 10.1038/s41392-017-0005-2 29682330 PMC5908807

[18] Serafimidis I, Rodriguez-Aznar E, Lesche M, Yoshioka K, Takuwa Y, Dahl A, et al Pancreas lineage allocation and specification are regulated by sphingosine-1-phosphate signalling. PLoS Biology. 2017;15(3):e2000949 10.1371/journal.pbio.2000949 PMC5331964. 28248965 PMC5331964

[19] Yu F-X, Guan K-L. The Hippo pathway: regulators and regulations. Genes Dev. 2013;27(4):355–71. 10.1101/gad.210773.112 23431053 PMC3589553

[20] Moroishi T, Hansen CG, Guan K-L. The emerging roles of YAP and TAZ in cancer. Nat Rev Cancer. 2015;15(2):73–9. 10.1038/nrc3876 25592648 PMC4562315

[21] Straßburger K, Tiebe M, Pinna F, Breuhahn K, Teleman AA. Insulin/IGF signaling drives cell proliferation in part via Yorkie/YAP. Dev Biol. 2012;367(2):187–96. 10.1016/j.ydbio.2012.05.008 22609549

[22] Wang J, Sinnett-Smith J, Stevens JV, Young SH, Rozengurt E. Biphasic Regulation of Yes-associated Protein (YAP) Cellular Localization, Phosphorylation, and Activity by G Protein-coupled Receptor Agonists in Intestinal Epithelial Cells: A NOVEL ROLE FOR PROTEIN KINASE D (PKD). J Biol Chem. 2016;291(34):17988–8005. 10.1074/jbc.M115.711275 27369082 PMC5016186

[23] Benham-Pyle BW, Pruitt BL, Nelson WJ. Mechanical strain induces E-cadherin–dependent Yap1 and β-catenin activation to drive cell cycle entry. Science. 2015;348(6238):1024–7. 10.1126/science.aaa4559 26023140 PMC4572847

[24] Kapoor A, Yao W, Ying H, Hua S, Liewen A, Wang Q, et al Yap1 Activation Enables Bypass of Oncogenic Kras Addiction in Pancreatic Cancer. Cell. 2014;158(1):185–97. 10.1016/j.cell.2014.06.003 24954535 PMC4109295

[25] Morvaridi S, Dhall D, Greene MI, Pandol SJ, Wang Q. Role of YAP and TAZ in pancreatic ductal adenocarcinoma and in stellate cells associated with cancer and chronic pancreatitis. Sci Rep. 2015;5:16759 10.1038/srep16759 http://www.nature.com/articles/srep16759#supplementary-information 26567630 PMC4645184

[26] Yang S, Zhang L, Purohit V, Shukla SK, Chen X, Yu F, et al Active YAP promotes pancreatic cancer cell motility, invasion and tumorigenesis in a mitotic phosphorylation-dependent manner through LPAR3. Oncotarget. 2015;6(34):36019–31. PMC4742158. 10.18632/oncotarget.5935 26440309 PMC4742158

[27] Murakami S, Shahbazian D, Surana R, Zhang W, Chen H, Graham GT, et al Yes-associated protein mediates immune reprogramming in pancreatic ductal adenocarcinoma. Oncogene. 2017;36(9):1232–44. 10.1038/onc.2016.288 27546622 PMC5322249

[28] Freed-Pastor William A, Mizuno H, Zhao X, Langerød A, Moon S-H, Rodriguez-Barrueco R, et al Mutant p53 Disrupts Mammary Tissue Architecture via the Mevalonate Pathway. Cell. 2012;148(1–2):244–58. 10.1016/j.cell.2011.12.017 22265415 PMC3511889

[29] Clendening JW, Pandyra A, Boutros PC, Ghamrasni SE, Khosravi F, Trentin GA, et al Dysregulation of the mevalonate pathway promotes transformation. Proc Natl Acad Sci U S A. 2010;107(34):15051–6. 10.1073/pnas.0910258107 20696928 PMC2930553

[30] Kuzu OF, Noory MA, Robertson GP. The Role of Cholesterol in Cancer. Cancer Res. 2016;76(8):2063–70. 10.1158/0008-5472.CAN-15-2613 27197250 PMC5813477

[31] Deng Y-Z, Cai Z, Shi S, Jiang H, Shang Y-R, Ma N, et al Cilia loss sensitizes cells to transformation by activating the mevalonate pathway. J Exp Med. 2018;215(1):177–95. 10.1084/jem.20170399 PMC5748847. 29237705 PMC5748847

[32] Gazzerro P, Proto MC, Gangemi G, Malfitano AM, Ciaglia E, Pisanti S, et al Pharmacological Actions of Statins: A Critical Appraisal in the Management of Cancer. Pharmacol Rev. 2012;64(1):102–46. 10.1124/pr.111.004994 22106090

[33] Clendening JW, Penn LZ. Targeting tumor cell metabolism with statins. Oncogene. 2012;31(48):4967–78. 10.1038/onc.2012.6 22310279

[34] Gruenbacher G, Thurnher M. Mevalonate Metabolism in Immuno-Oncology. Front Immunol. 2017;8:1714 10.3389/fimmu.2017.01714 PMC5717006. 29250078 PMC5717006

[35] Sorrentino G, Ruggeri N, Specchia V, Cordenonsi M, Mano M, Dupont S, et al Metabolic control of YAP and TAZ by the mevalonate pathway. Nat Cell Biol. 2014;16(4):357–66. 10.1038/ncb2936 http://www.nature.com/ncb/journal/v16/n4/abs/ncb2936.html#supplementary-information 24658687

[36] Santinon G, Pocaterra A, Dupont S. Control of YAP/TAZ Activity by Metabolic and Nutrient-Sensing Pathways. Trends Cell Biol. 2016;26(4):289–99. 10.1016/j.tcb.2015.11.004 26750334

[37] Wang Z, Wu Y, Wang H, Zhang Y, Mei L, Fang X, et al Interplay of mevalonate and Hippo pathways regulates RHAMM transcription via YAP to modulate breast cancer cell motility. Proc Natl Acad Sci U S A. 2014;111(1):E89–E98. 10.1073/pnas.1319190110 24367099 PMC3890879

[38] Jeon CY, Pandol SJ, Wu B, Cook-Wiens G, Gottlieb RA, Merz NB, et al The Association of Statin Use after Cancer Diagnosis with Survival in Pancreatic Cancer Patients: A SEER-Medicare Analysis. PLoS ONE. 2015;10(4):e0121783 10.1371/journal.pone.0121783 25830309 PMC4382214

[39] Wu BU, Chang J, Jeon CY, Pandol SJ, Huang B, Ngor EW, et al Impact of Statin Use on Survival in Patients Undergoing Resection for Early-Stage Pancreatic Cancer. Am J Gastroenterol. 2015;110(8):1233–9. 10.1038/ajg.2015.217 26195180 PMC4877304

[40] Chen M-J, Tsan Y-T, Liou J-M, Lee Y-C, Wu M-S, Chiu H-M, et al Statins and the risk of pancreatic cancer in Type 2 diabetic patients—A population-based cohort study. Int J Cancer. 2016;138(3):594–603. 10.1002/ijc.29813 26296262

[41] Walker EJ, Ko AH, Holly EA, Bracci PM. Statin use and risk of pancreatic cancer: Results from a large, clinic-based case-control study. Cancer. 2015;121(8):1287–94. 10.1002/cncr.29256 25649483 PMC4393339

[42] Carey FJ, Little MW, Pugh TFG, Ndokera R, Ing H, Clark A, et al The Differential Effects of Statins on the Risk of Developing Pancreatic Cancer: A Case–Control Study in Two Centres in the United Kingdom. Dig Dis Sci. 2013;58(11):3308–12. 10.1007/s10620-013-2778-7 23864194

[43] Lee HS, Lee SH, Lee HJ, Chung MJ, Park JY, Park SW, et al Statin Use and Its Impact on Survival in Pancreatic Cancer Patients. Medicine. 2016;95(19):e3607 10.1097/MD.0000000000003607 PMC4902509. 27175667 PMC4902509

[44] Huang BZ, Chang JI, Li E, Xiang AH, Wu BU. Influence of Statins and Cholesterol on Mortality Among Patients With Pancreatic Cancer. J Natl Cancer Inst. 2017;109(5):djw275–djw. 10.1093/jnci/djw275 28040693

[45] Hamada T, Khalaf N, Yuan C, Morales-Oyarvide V, Babic A, Nowak JA, et al Pre-diagnosis Use of Statins Associates With Increased Survival Times of Patients With Pancreatic Cancer. Clin Gastroenterol Hepatol. 2018 10.1016/j.cgh.2018.02.022 PMC6056316 29474971

[46] Archibugi L, Piciucchi M, Stigliano S, Valente R, Zerboni G, Barucca V, et al Exclusive and Combined Use of Statins and Aspirin and the Risk of Pancreatic Cancer: a Case-Control Study. Sci Rep. 2017;7(1):13024 10.1038/s41598-017-13430-z 29026148 PMC5638859

[47] Jian-Yu E, Graber JM, Lu S-E, Lin Y, Lu-Yao G, Tan X-L. Effect of Metformin and Statin Use on Survival in Pancreatic Cancer Patients: a Systematic Literature Review and Meta-analysis. Curr Med Chem. 2018;25(22):2595–607. 10.2174/0929867324666170412145232 . 28403788 PMC5638687

[48] Mei Z, Liang M, Li L, Zhang Y, Wang Q, Yang W. Effects of statins on cancer mortality and progression: A systematic review and meta-analysis of 95 cohorts including 1,111,407 individuals. Int J Cancer. 2017;140(5):1068–81. 10.1002/ijc.30526 27859151

[49] Mohammed A, Qian L, Janakiram NB, Lightfoot S, Steele VE, Rao CV. Atorvastatin delays progression of pancreatic lesions to carcinoma by regulating PI3/AKT signaling in p48Cre/+ LSL-KrasG12D/+ mice. Int J Cancer. 2012;131(8):1951–62. 10.1002/ijc.27456 22287227 PMC3376252

[50] Chang H-H, Eibl G, Rozengurt E. Models and Mechanisms of High-Fat Diet (HFD) Promotion of Pancreatic Cancer In: Berger NA, editor. Murine Models, Energy Balance, and Cancer. Energy Balance and Cancer. 10: Springer International Publishing; 2015 p. 197–215.

[51] Funahashi H, Satake M, Dawson D, Huynh NA, Reber HA, Hines OJ, et al Delayed progression of pancreatic intraepithelial neoplasia in a conditional Kras(G12D) mouse model by a selective cyclooxygenase-2 inhibitor. Cancer Res. 2007;67(15):7068–71. 10.1158/0008-5472.CAN-07-0970 . 17652141

[52] Kisfalvi K, Guha S, Rozengurt E. Neurotensin and EGF induce synergistic stimulation of DNA synthesis by increasing the duration of ERK signaling in ductal pancreatic cancer cells. J Cell Physiol. 2005;202(3):880–90. 10.1002/jcp.20187 . 15389644

[53] Kisfalvi K, Rey O, Young SH, Sinnett-Smith J, Rozengurt E. Insulin Potentiates Ca2+ Signaling and Phosphatidylinositol 4,5-Bisphosphate Hydrolysis Induced by Gq Protein-Coupled Receptor Agonists through an mTOR-Dependent Pathway. Endocrinology. 2007;148:3246–57. 10.1210/en.2006-1711 . 17379645

[54] Kisfalvi K, Eibl G, Sinnett-Smith J, Rozengurt E. Metformin disrupts crosstalk between G protein-coupled receptor and insulin receptor signaling systems and inhibits pancreatic cancer growth. Cancer Res. 2009;69(16):6539–45. 10.1158/0008-5472.CAN-09-0418 . 19679549 PMC2753241

[55] Young SH, Rozengurt E. Crosstalk between insulin receptor and G protein-coupled receptor signaling systems leads to Ca(2+) oscillations in pancreatic cancer PANC-1 cells. Biochem Biophys Res Commun. 2010;401(1):154–8. 10.1016/j.bbrc.2010.09.036 . 20849815 PMC2952715

[56] Rozengurt E, Sinnett-Smith J, Kisfalvi K. Crosstalk between Insulin/Insulin-like Growth Factor-1 Receptors and G Protein-Coupled Receptor Signaling Systems: A Novel Target for the Antidiabetic Drug Metformin in Pancreatic Cancer. Clin Cancer Res. 2010;16:2505–11. 10.1158/1078-0432.CCR-09-2229 20388847 PMC2862089

[57] Rozengurt E. Mechanistic target of rapamycin (mTOR): a point of convergence in the action of insulin/IGF-1 and G protein-coupled receptor agonists in pancreatic cancer cells. Front Physiol. 2014;5:357 10.3389/fphys.2014.00357 PMC4171984. 25295009 PMC4171984

[58] Hao F, Xu Q, Zhao Y, Stevens JV, Young SH, Sinnett-Smith J, et al Insulin Receptor and GPCR Crosstalk Stimulates YAP via PI3K and PKD in Pancreatic Cancer Cells. Mol Cancer Res. 2017;15(7):929–41. 10.1158/1541-7786.MCR-17-0023 28360038 PMC5645013

[59] Gbelcová H, Rimpelová S, Ruml T, Fenclová M, Kosek V, Hajšlová J, et al Variability in statin-induced changes in gene expression profiles of pancreatic cancer. Sci Rep. 2017;7:44219 10.1038/srep44219 PMC5343581. 28276528 PMC5343581

[60] Beckwitt CH, Shiraha K, Wells A. Lipophilic statins limit cancer cell growth and survival, via involvement of Akt signaling. PloS ONE. 2018;13(5):e0197422–e. 10.1371/journal.pone.0197422 . 29763460 PMC5953490

[61] Soares HP, Ming M, Mellon M, Young SH, Han L, Sinnet-Smith J, et al Dual PI3K/mTOR Inhibitors Induce Rapid Overactivation of the MEK/ERK Pathway in Human Pancreatic Cancer Cells through Suppression of mTORC2. Mol Cancer Ther. 2015;14(4):1014–23. 10.1158/1535-7163.MCT-14-0669 25673820 PMC4394038

[62] Uhlen M, Zhang C, Lee S, Sjöstedt E, Fagerberg L, Bidkhori G, et al A pathology atlas of the human cancer transcriptome. Science. 2017;357(6352). 10.1126/science.aan2507 28818916

[63] Parrales A, Ranjan A, Iyer Swathi V, Padhye S, Weir Scott J, Roy A, et al DNAJA1 controls the fate of misfolded mutant p53 through the mevalonate pathway. Nat Cell Biol. 2016;18:1233–43. 10.1038/ncb3427 https://www.nature.com/articles/ncb3427#supplementary-information 27775703 PMC5340314

[64] Zanconato F, Cordenonsi M, Piccolo S. YAP/TAZ at the Roots of Cancer. Cancer Cell. 2016;29(6):783–803. 10.1016/j.ccell.2016.05.005 27300434 PMC6186419

[65] Yang S, Zhang L, Purohit V, Shukla SK, Chen X, Yu F, et al Active YAP promotes pancreatic cancer cell motility, invasion and tumorigenesis in a mitotic phosphorylation-dependent manner through LPAR3 2015 10.18632/oncotarget.5935 26440309 PMC4742158

[66] Xie D, Cui J, Xia T, Jia Z, Wang L, Wei W, et al Hippo transducer TAZ promotes epithelial mesenchymal transition and supports pancreatic cancer progression 2015 10.18632/oncotarget.5772 26416426 PMC4742153

[67] Plouffe SW, Lin KC, Moore JL, Tan FE, Ma S, Ye Z, et al The Hippo pathway effector proteins YAP and TAZ have both distinct and overlapping functions in the cell. J Biol Chem. 2018;293(28):11230–40. 10.1074/jbc.RA118.002715 29802201 PMC6052207

[68] Panciera T, Azzolin L, Cordenonsi M, Piccolo S. Mechanobiology of YAP and TAZ in physiology and disease. Nat Rev Mol Cell Biol. 2017;18(12):758–70. 10.1038/nrm.2017.87 PMC6192510. 28951564 PMC6192510

[69] Das A, Fischer RS, Pan D, Waterman CM. YAP Nuclear Localization in the Absence of Cell-Cell Contact Is Mediated by a Filamentous Actin-dependent, Myosin II- and Phospho-YAP-independent Pathway during Extracellular Matrix Mechanosensing. J Biol Chem. 2016;291(12):6096–110. 10.1074/jbc.M115.708313 PMC4813550. 26757814 PMC4813550

[70] Etienne-Manneville S, Hall A. Rho GTPases in cell biology. Nature. 2002;420(6916):629–35. 10.1038/nature01148 . 12478284

[71] Elosegui-Artola A, Andreu I, Beedle AEM, Lezamiz A, Uroz M, Kosmalska AJ, et al Force Triggers YAP Nuclear Entry by Regulating Transport across Nuclear Pores. Cell. 2017;171(6):1397–410.e14. 10.1016/j.cell.2017.10.008 29107331

[72] Dobrokhotov O, Samsonov M, Sokabe M, Hirata H. Mechanoregulation and pathology of YAP/TAZ via Hippo and non-Hippo mechanisms. Clin Transl Med. 2018;7:23 10.1186/s40169-018-0202-9 PMC6087706. 30101371 PMC6087706

[73] Hamada T, Khalaf N, Yuan C, Babic A, Morales-Oyarvide V, Qian ZR, et al Statin use and pancreatic cancer risk in two prospective cohort studies. J Gastroenterol. 2018;53(8):959–66. 10.1007/s00535-018-1430-x 29362938 PMC7609961

[74] Hamada T, Khalaf N, Yuan C, Morales-Oyarvide V, Babic A, Nowak JA, et al Prediagnosis Use of Statins Associates With Increased Survival Times of Patients With Pancreatic Cancer. Clin Gastroenterol Hepatol. 2018;16(8):1300–6.e3. 10.1016/j.cgh.2018.02.022 29474971 PMC6056316

