# Supplementary figures and images for: Lipophilic statins inhibit YAP nuclear localization, co-activator activity and colony formation in pancreatic cancer cells and prevent the initial stages of pancreatic ductal adenocarcinoma in KrasG12D mice
Source: PLoS One. 2019 May 17;14(5):e0216603. doi: 10.1371/journal.pone.0216603 (PMC6524808; doi:10.1371/journal.pone.0216603)

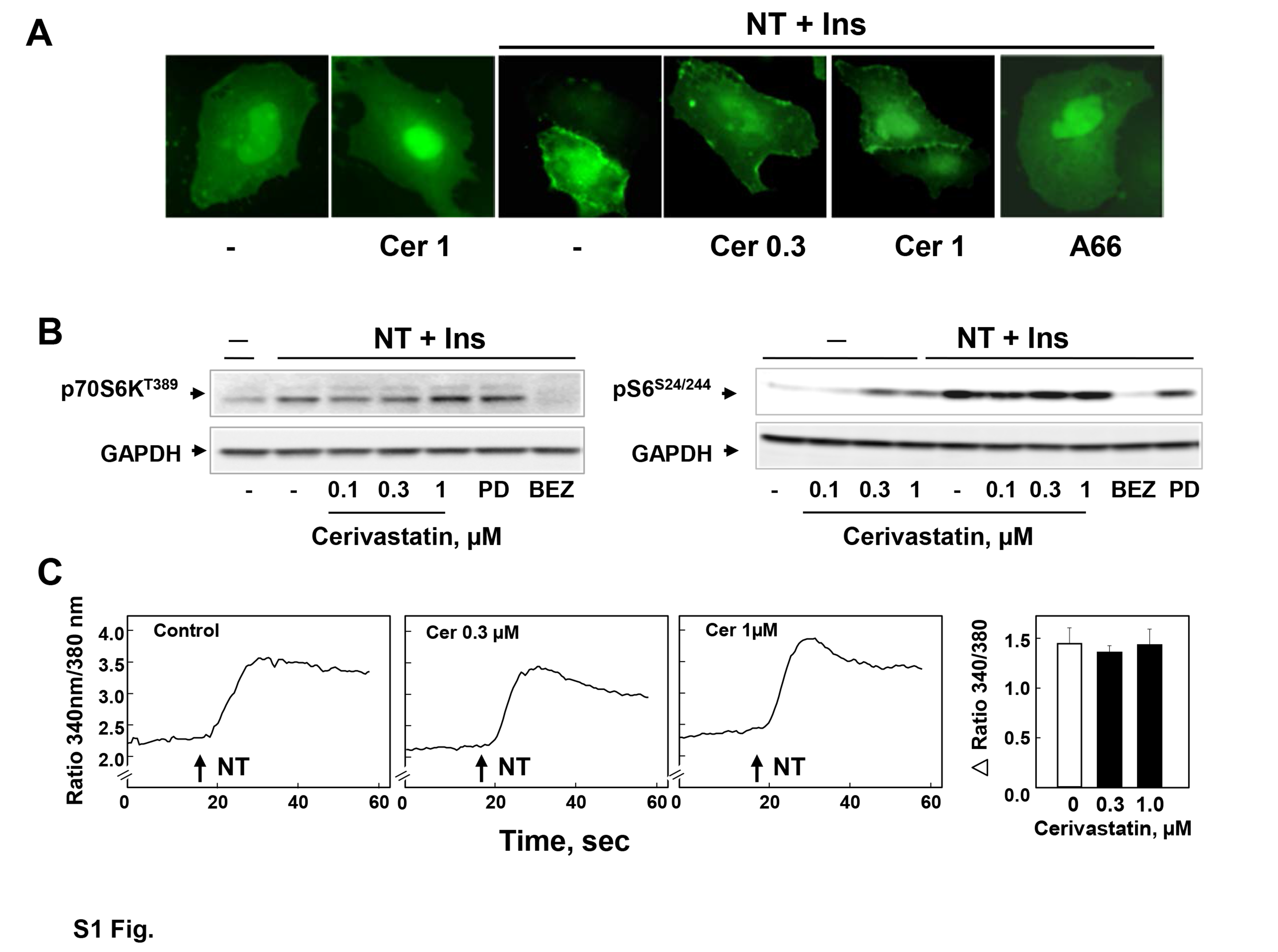

Supplement: S1 Fig — A: PANC-1 cells were transiently transfected with a plasmid encoding a fusion protein between GFP and the PH domain of AKT (AKT-PH-GFP). After 24h, the cultures were incubated in DMEM without or with cerivastatin at the indicated concentrations for 18h prior to stimulation with 5 nM neurotensin and 10 ng/ml insulin. Other cultures weretreated with the class I p110α specific inhibitor A66 at 10 μM, tested as a positive control The intracellular distribution of AKT-PH-GFP was monitored under a fluorescence microscope. The selected cells displayed in the figures were representative of 90% of the population of GFP-positive cells. B: PANC-1 cells were treated for 24 h either in the absence or presence of cerivastatin (Cer) at the indicated concentrations for 24h. Other cultures were incubated for 2h with the either the MEK inhibitor PD0325901 (1μM, PD) or the dual PI3K/mTOR inhibitor NPV-BEZ235 (1μM, BEZ). All cultures were then stimulated with 5 nM neurotensin and 10 ng/ml insulin (NT+Ins) for 30 min as indicated, and lysed with SDS–PAGE sample buffer. The samples were analyzed by SDS-PAGE and immunoblotting with phospho-p70 S6 KinaseThr-389 and phospho-S6 Ribosomal Protein Ser-240/244. Equal loading was verified by immunoblotting with GAPDH antibody.Similar results were obtained in 2 independent experiments. C: PANC-1 cells were incubated without or with cerivastatin at the indicated concentrations for 18h prior to stimulation with 5 nM neurotensin. Intracellular [Ca2+]i was monitored as described in Materials and Methods. (TIF) [file pone.0216603.s001.TIF]

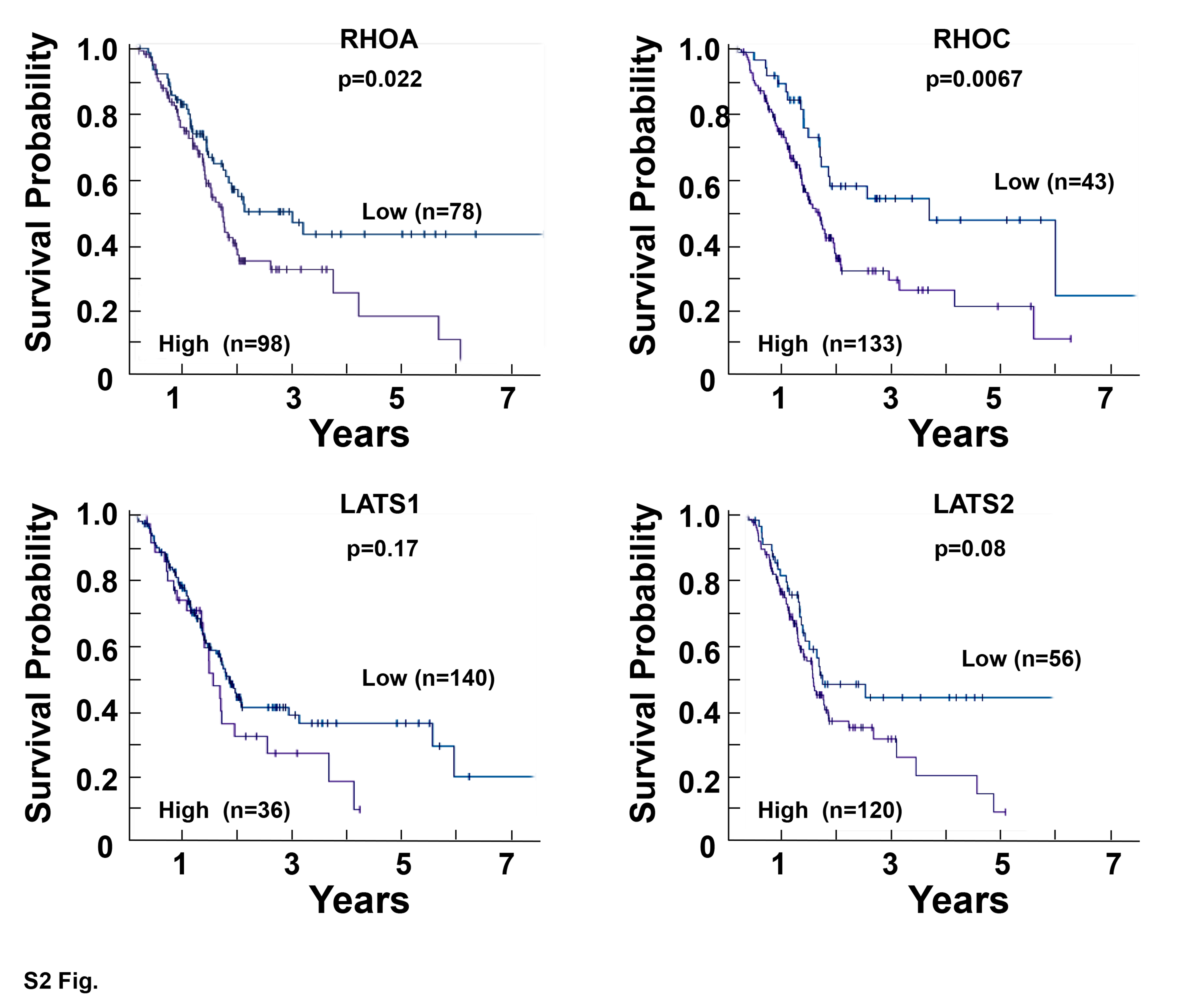

Supplement: S2 Fig — Images were reproduced from the Human Protein Atlas (version 17) available from www.proteinatlas.org The link is: http://www.proteinatlas.org/ENSG00000137693YAP1/pathology/tissue/pancreatic+cancerS1 (TIF) [file pone.0216603.s002.TIF]

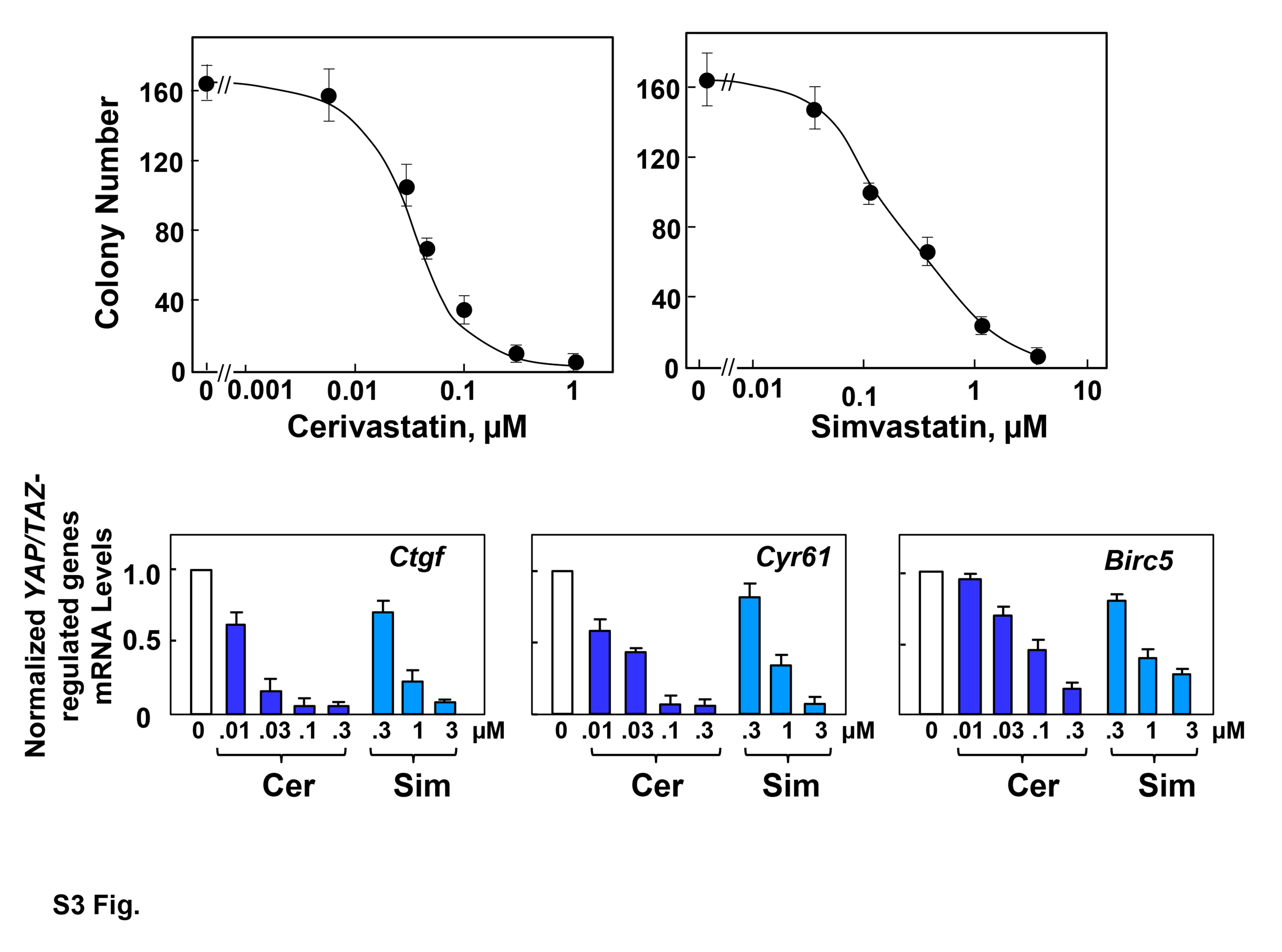

Supplement: S3 Fig — A, KPC cells were incubated for 6 days with various concentrations of cerivastatin or simvastatin, as indicated. The bars represent the number of colonies (mean ± SEM; n = 4 dishes per condition). B, KPC cells were incubated either in absence or presence of cerivastatin (Cer) or simvastatin (Sim) at the indicated concentrations. Statins were added 1 day after plating and the incubation continued for 24 h. RNA was then isolated and the relative levels (n = 3) of CTGF, CYR61 and BIRC5 mRNA compared with 18s mRNA were measured by RT-qPCR. Data are presented as mean ± SEM. Similar results were obtained in 3 independent experiments. (TIF) [file pone.0216603.s003.TIF]
